# Z-Disk-Associated Plectin (Isoform 1d): Spatial Arrangement, Interaction Partners, and Role in Filamin C Homeostasis

**DOI:** 10.3390/cells12091259

**Published:** 2023-04-26

**Authors:** Lilli Winter, Ilona Staszewska-Daca, Stefan Zittrich, Fatiha Elhamine, Michaela M. Zrelski, Katy Schmidt, Irmgard Fischer, Christian Jüngst, Astrid Schauss, Wolfgang H. Goldmann, Robert Stehle, Gerhard Wiche

**Affiliations:** 1 Department of Biochemistry and Cell Biology, Max Perutz Laboratories, University of Vienna, 1030 Vienna, Austria; lilli.winter@meduniwien.ac.at (L.W.); daca.ilona@gmail.com (I.S.-D.); irmgard.fischer@univie.ac.at (I.F.); 2 Division of Cell and Developmental Biology, Center for Anatomy and Cell Biology, Medical University of Vienna, 1090 Vienna, Austria; michaela.zrelski@meduniwien.ac.at (M.M.Z.); katy.schmidt@univie.ac.at (K.S.); 3 Institute of Vegetative Physiology, Medical Faculty, University of Cologne, 50931 Cologne, Germany; stefan.zittrich@uni-koeln.de (S.Z.); fatiha.elhamine@uni-koeln.de (F.E.); robert.stehle@uni-koeln.de (R.S.); 4 Core Facility for Cell Imaging & Ultrastructure Research (CIUS), University of Vienna, 1030 Vienna, Austria; 5 CECAD Imaging Facility, CECAD Forschungszentrum Cologne, 50931 Cologne, Germany; cjuengst@uni-koeln.de (C.J.); aschauss@uni-koeln.de (A.S.); 6 Department of Physics, Center for Medical Physics and Technology, Friedrich-Alexander-University Erlangen-Nuremberg, 91052 Erlangen, Germany; wolfgang.goldmann@fau.de

**Keywords:** plectin, skeletal muscle, myofibril, cytolinker, desmin intermediate filaments, chaperone-assisted selective autophagy

## Abstract

Plectin, a highly versatile cytolinker protein, is crucial for myofiber integrity and function. Accordingly, mutations in the human gene (*PLEC*) cause several rare diseases, denoted as plectinopathies, with most of them associated with progressive muscle weakness. Of several plectin isoforms expressed in skeletal muscle and the heart, P1d is the only isoform expressed exclusively in these tissues. Using high-resolution stimulated emission depletion (STED) microscopy, here we show that plectin is located within the gaps between individual α-actinin-positive Z-disks, recruiting and bridging them to desmin intermediate filaments (IFs). Loss of plectin in myofibril bundles led to a complete loss of desmin IFs. Loss of Z-disk-associated plectin isoform P1d led to disorganization of muscle fibers and slower relaxation of myofibrils upon mechanical strain, in line with an observed inhomogeneity of muscle ultrastructure. In addition to binding to α-actinin and thereby providing structural support, P1d forms a scaffolding platform for the chaperone-assisted selective autophagy machinery (CASA) by directly interacting with HSC70 and synpo2. In isoform-specific knockout (P1d-KO) mouse muscle and mechanically stretched plectin-deficient myoblasts, we found high levels of undigested filamin C, a bona fide substrate of CASA. Similarly, subjecting P1d-KO mice to forced swim tests led to accumulation of filamin C aggregates in myofibers, highlighting a specific role of P1d in tension-induced proteolysis activated upon high loads of physical exercise and muscle contraction.

## 1. Introduction

Striated muscles are elaborately organized machines designated for contraction, and their highly textured structure is a prerequisite for directed force development. Sarcomeres, the smallest functional units of muscle contraction, comprise precisely organized filament systems including thin (actin) and thick (myosin) filaments, titin, and nebulin [1], which build up the myofibrillar apparatus. The extrasarcomeric cytoskeleton connects the sarcomeres with the sarcolemma and the extracellular matrix through linking the Z-disks to the sub-membrane cytoskeleton, thereby ensuring the transmission of the forces produced by the sarcomeres. Desmin intermediate filaments (IFs), constituting the principal component of the extrasarcomeric cytoskeleton, form a three-dimensional scaffold extending from the nuclear envelope to the sarcolemma and tethering membranous organelles, such as mitochondria and the sarcoplasmic reticulum [2]. In the heart, desmin IFs are also particularly abundant at the intercalated disks, thereby providing structural and mechanical support to cardiac tissue [2].

Plectin, a multi-modular cytolinker protein of exceptionally large size (>500 kDa), orchestrates and functionally organizes various cytoskeletal networks, and thereby contributes to fundamental biomechanical properties of stress-bearing tissues, such as skeletal muscle [3]. Acting as a multi-functional linker protein and signaling scaffold, plectin possesses binding sites for all types of IF subunit proteins, enabling it to anchor IFs to sites of strategic importance for the organization and performance of cells [4]. In addition, plectin harbors a functional actin-binding domain (ABD), binds to microtubule-associated proteins (MAPs), and interacts with transmembrane receptors, proteins of the sub-membrane cytoskeleton, components of the nuclear envelope, and several kinases with known roles in the migration, proliferation, and energy metabolism of cells [5,6]. In the absence of plectin, mechanically stressed tissues, such as skin and muscle, lose their structural integrity, as observed in patients suffering from plectinopathies and in plectin-deficient mice [7,8,9]. Most mutations in the human plectin gene (*PLEC*) on chromosome 8q24 lead to epidermolysis bullosa simplex with muscular dystrophy (EBS-MD, MIM #226670), an autosomal recessive skin blistering disorder associated with progressive muscle weakness [7,8]. In addition, plectin mutations have been shown to cause EBS-MD with a myasthenic syndrome (EBS-MD-MyS), EBS with pyloric atresia (EBS-PA, MIM #612138), limb-girdle muscular dystrophy (LGMDR17, MIM #613723), EBS with nail dystrophy (EBSND, MIM #616487), or the autosomal dominant variant EBS-Ogna (MIM #131950) [3,9]. Emerging cardiac disease manifestations including left ventricular hypertrophy, atrial fibrillation, ventricular non-compaction myopathy, dilated cardiomyopathy, and life-threatening episodes of arrhythmias emphasize the pathological consequences of *PLEC* mutations [3]. Taken together, plectinopathies have emerged as complex multi-systemic disorders, affecting primarily tissues exposed to great mechanical stress, such as skin, skeletal muscle, and the heart, but exhibiting a variety of additional symptoms and disease manifestations.

Plectin’s versatility is in part due to an unusual transcript diversity, where complex splicing events in its N-terminal region give rise to multiple alternatively spliced isoforms containing distinct first exons [4,5]. Individual plectin isoforms are differentially expressed in various types of cells and tissues in specific combinations and proportions, where they function as universal IF-docking sites [5,6], thereby substantially contributing to fundamental biomechanical properties of stress-bearing tissues. In striated muscle tissue, plectin is predominantly found at the level of Z-disks, where it co-localizes with desmin IFs. The four most prominently expressed isoforms of plectin in muscle are P1d, P1f, P1b, and P1, which are accountable for myofiber integrity by anchoring desmin IFs to Z-disks, costameres, mitochondria, and the nuclear/sarcoplasmic reticulum membrane system, respectively [10,11]. With an isoform-specific N-terminal sequence that is just five amino acid residues long, P1d is the only isoform that is exclusively expressed in skeletal muscle and heart, as identified by plectin transcript profiling [4,5], and is specifically targeted to Z-disks, as demonstrated in differentiated myotubes and muscle fibers [12]. Consequently, when skeletal muscle fibers, isolated from isoform-specific P1d-knockout (P1d-KO) mice (lacking just P1d, but expressing the other isoforms [11]) were immunolabeled using pan-plectin-antibodies (reactive with all plectin isoforms), the Z-disk-specific labeling was lost, and only sarcolemmal staining and dotty remnants in the interior of the fiber became evident. These results indicated that in P1d-KO muscles, P1 and P1f were still preserved in the peripheral, perinuclear, and costameric regions, while Z-disk-associated P1d was absent [11]. Accordingly, accumulation of desmin-positive protein aggregates was noted in the interior, but not in peripheral areas of P1d-KO muscle fibers. Moreover, P1d-KO muscles disclosed extensive misalignment of myofibrils and disorientation of Z-disks, thereby closely resembling the pathology of muscles completely devoid of plectin, such as those from conditional, skeletal muscle-specific plectin knockout (MCK-Cre/cKO) mice [11]. However, compared to the latter, the desmin IF network underneath the sarcolemma appeared unaffected in P1d-KO teased fibers, suggesting that P1f still maintains the organization of the costameres [11]. Finally, when P1d-GFP fusion proteins were expressed in differentiated plectin-deficient myotubes, a cell model closely mimicking the EBS-MD-associated skeletal muscle pathology by displaying impaired Z-disk alignment and desmin IF network collapse, they fully restored sarcomere formation and recruited desmin IFs to the Z-disks [13]. In contrast, forced expression of any of the other plectin isoforms typical for skeletal muscle (P1, P1b, or P1f) showed no such effect [13]. Taken together, these experiments highlighted that the striking sarcomere pathology of EBS-MD muscle is primarily due to a loss of function of P1d, the isoform which tethers myofibrils to the desmin IF network at the level of Z-disks.

However, important questions regarding mechanistic aspects of Z-disk-associated plectin and its functionality as well as the pathology arising from plectin deficiency remained unanswered. For instance, neither the targeting mechanisms nor the spatial arrangement of P1d and desmin IFs at Z-disks have been investigated so far, nor is anything known about the biomechanical consequences of P1d-deficiency for individual myofibrils. Moreover, it can be assumed that as a variant of a multifunctional cytolinker protein with a well-established role as versatile cytoplasmic signaling platform [5,6], P1d exerts important functions beyond IF anchorage at Z disks in muscle fibers. Addressing these and other contextually relevant issues in the present study, we used high-resolution stimulated emission depletion (STED) microscopy to unravel the spatial arrangement of plectin and desmin networks at the level of individual Z-disks, and yeast two-hybrid (Y2H) screening to identify isoform-specific P1d interaction partners associated with Z-disks. In addition, we assessed the consequences of site-specific (Z-disk-restricted) plectin deficiency on muscle function ex vivo and in vivo by subjecting isoform P1d-specific knockout (P1d-KO) mice, and derivatives thereof, to diverse phenotypic analyses. Among the physiological parameters found to be affected by P1d deficiency were the relaxation kinetics of myofibrillar sarcomeres and the homeostasis of the chaperone-assisted selective autophagy (CASA) proteolytic degradation machinery and its major substrate, the actin-crosslinking protein filamin C. On the organismal level, physical exercise of P1d-KO mice led to myofibrillar lesions, early exhaustion, and lethality. In all, our experiments unequivocally reveal an important role of P1d in the maintenance of muscle structure and provide new insight(s) into the molecular mechanisms underlying plectinopathy-related muscle weakness.

## 2. Materials and Methods

### 2.1. Animals

All experiments involving animals were performed according to the Austrian Federal Government laws and regulations. MCK-Cre/cKO, P1d-KO, and P1b-KO mice have been published [11,14].

Myofibril bundles for mechanical experiments, as well as bundles and cryosections for STED microscopy, were prepared from MCK-Cre/cKO, P1d KO, or corresponding wild-type mice that were sacrificed by cervical dislocation according to the guidelines and as approved by the ethical committee of the Landesamt für Natur, Umwelt und Verbraucherschutz Nordrhein-Westfalen (LANUV).

Behavioral assessments were approved by the Federal Ministry for Science, Research and Economy, Vienna, Austria (BMWFW-66.006/0005-WF/II3b/2014) and performed in cooperation with the Preclinical Phenotyping Facility at Vienna Biocenter Core Facilities (VBCF), a member of Vienna Biocenter (VBC), Austria. During the forced swim test [15], mice were placed into a round tank filled with pre-warmed water (30–32 °C). Endurance exercises, as described in [16], were performed according to a ramp protocol starting with 20 min swimming daily, with 10 min increase each day, until 90 min were reached. Mice were exercised daily for two weeks. Animals were continuously observed during the swim test, and any animal that sank below the surface was removed from the water immediately. Mice exhibiting signs of exhaustion during the training session were removed from the water and allowed to rest for 3 min in their home cage before resuming the session. After a workload application, mice were allowed to dry in a warm environment after removal from the water.

### 2.2. Antibodies

Primary antibodies used for immunofluorescence microscopy (IFM) and/or immunoblotting (IB) are listed in Table 1. For IFM, primary antibodies were used in combination with goat-anti-mouse IgG Alexa 488, donkey-anti-mouse IgG Rhodamine Red, goat-anti-rat IgG Alexa 488, goat-anti-rabbit IgG Alexa 488, and donkey-anti-rabbit IgG Rhodamine Red at dilutions of 1:500 (all from Jackson ImmunoResearch Laboratories, Cambridge, UK). For IB analyses, HRP-conjugated secondary antibodies were used at dilutions of 1:5000–1:10,000 (Jackson ImmunoResearch Laboratories). Pan-plectin antibodies (rabbit antiserum #9 [17]) were used for immunoprecipitation. For STED microscopy, primary antibodies were used in combination with the goat-anti-mouse IgG Atto 647N, goat-anti-rabbit IgG Abberior STAR 580, goat-anti-rabbit IgG (H+L) Alexa 488, goat-anti-rabbit IgG (H+L) Alexa 647, and goat-anti-mouse IgG (H+L) Alexa 647 or Zenon anti-mouse IgG_1_ 488 labelling kits (all from Sigma-Aldrich, St. Louis, MO, USA).

### 2.3. Preparation of Myofibrils

Muscle strips (0.2–0.3 mm in diameter) were dissected from psoas and soleus muscle and incubated for 2 h in skinning solution (5 mM K-phosphate, 5 mM Na-azide, 3 mM Mg-acetate, 5 mM K_2_EGTA, 3 mM Na_2_ATP (including 3 mM MgCl_2_ and 6 mM KOH), 47 mM Na_2_CrP, 2 mM DTT, 0.5 mM 4-(2-aminoethyl)-benzenesulfonylfluoride-HCl, 10 μM leupeptine, 10 μM antipaine, and 5 mg/mL aprotinine, adjusted to pH 6.8 at 20 °C) as described in [21], supplemented with 30 mM 2,3-butanedione monoxime (BDM) and 0.5% Triton X-100. The solution was replaced by a similar one without detergent and strips, and stored for up to 3 days at 0 °C. For long-term storage, strips were successively incubated (for 30 min each), in skinning solutions containing 0.5 M, 1.0 M, 1.5 M, and 2.0 M sucrose, then rapidly frozen in liquid propane and transferred into liquid nitrogen [22]. Immediately before mechanical or immunostaining experiments, cryo-conserved strips were thawed and consecutively incubated in skinning solutions containing 2 M, 1.5 M, 1.0 M, 0.5 M, and 0 M sucrose, and then homogenized at 4 °C for 5–10 s with a blender (Ultra-Turrax T25, Janke & Kunkel, Staufen, Germany) at maximum speed.

### 2.4. Microscopy

For STED microscopy of cryosections, wild-type mouse soleus muscles were dissected in HEPES-buffered, Krebs–Ringer solution, pinned at the tendons, and permeabilized in skinning solution with 1% Triton X-100, 30 mM BDM for 1 h. After washing, the muscle was frozen in liquid nitrogen and stored at −80 °C overnight. Cryosections (10 µm) were then cut, immediately fixed in 3% glyoxal, quenched with 100 mM NH_4_Cl, and permeabilized in PBS containing 0.1% Triton X-100 and 2.5% bovine serum albumin (BSA). Cryosections were immunolabeled with primary antibodies at dilutions of 1:100 (plectin) or 1:125 (desmin), and with secondary antibodies (Atto 647, Abberior 580) at a dilution of 1:75.

For STED microscopy of myofibril bundles, bundles were sedimented (in skinning solution) onto coverslips for 45 min at 0 °C. Specimens were blocked with 1% BSA and 3% goat serum and incubated with primary antibodies at dilutions 1:100–1:200 (plectin and desmin) and 1:800–1:1000 (α-actinin), and with secondary antibodies at dilutions of 1:500–1:1000. Bundles were immobilized on coverslips either prior to blocking by fixation with 3% paraformaldehyde for 4 min, or after immunolabeling by immersion with PBS containing 10 mM β-mercaptoethylamine.

Confocal and STED images were taken with a TCS SP8 gSTED microscope using a HSX PL Apo ×100/1.40 oil immersion objective (Leica Microsystems, Wetzlar, Germany). The two channels were acquired in a sequential mode. Deconvolution of the images was carried out using the Huygens Professional (Scientific Volume Imaging) software.

Thin sections (5 µm) were obtained from soleus muscle tissue that was frozen in isopentane cooled with liquid nitrogen. Teased muscle fibers were prepared and processed as described previously [11,12]. The staining procedure was performed using the M.O.M. Basic Kit (Vector Laboratories, Newark, CA, USA) according to the manufacturer’s instructions, as described in [13]. Nuclei were stained with Hoechst 33258 (Sigma-Aldrich), and samples were mounted in Mowiol 4-88 (Hoechst, Frankfurt, Germany). Confocal microscopy was performed using a fluorescence laser scanning microscope (LSM710, Zeiss, Oberkochen, Germany) equipped with Plan Apochromat 63 × 1.4 NA and 40 × 1.3 NA objective lenses. Images were recorded using the LSM710 module and the Zeiss ZEN software.

### 2.5. Transmission Electron Microscopy

Freshly dissected soleus muscles were fixed in 2% paraformaldehyde and 2.5% glutaraldehyde in 0.1 M cacodylate buffer. Specimens were washed three times and post-fixed in 1% OsO4 with 1.5% potassium hexacyanoferrate(II) followed by dehydration in an ascending ethanol series. After embedding in Epon resin, 50 nm ultrathin sections were cut with a Reichert-Jung ULTRACUT microtome and counterstained with lead citrate and uranyl acetate. Images were acquired using an FEI Tecnai20 electron microscope equipped with a 4 K Eagle-CCD camera, and then processed with Adobe Photoshop.

### 2.6. Force Measurements of Myofibrils

Force measurement were performed at 10 °C using an experimental setup described previously [21]. Myofibril bundles (diameters of 1.7–3.4 mm) were mounted in relaxing solution (pCa 8) between the tip of an atomic-force cantilever and the tip of a length-driving stiff tungsten needle. After mounting, the slack sarcomere length, overall length, and the diameter of the bundles were determined. The bundles were stretched to a sarcomere length of 2.45 µm and the passive force was determined in relaxing solution (pCa 7). Bundles were rapidly activated and relaxed by a rapid solution change (within 10 ms), and the force kinetics were recorded and thereafter analyzed as described previously [21,23]. Each bundle was first activated twice at pCa 4.5 to determine its active force and rate constants of Ca^2+^-induced and mechanically-induced force development before eccentric contraction. Then, the bundle was stretched in the third activation for 250 ms with a constant speed of 1 times its length per second, held for 500 ms at the stretched length, released with same speed to the original length, and then force and force kinetic parameters were determined after the eccentric contraction.

### 2.7. Yeast Two-Hybrid Screening

For the yeast two-hybrid screening, cDNAs encoding plectin fragments P1d-8(2α) and P1f-8 [10], as well as cDNAs encoding plectin fragments P1d-24(2α) or P1f-24 [24], were sub-cloned into a modified pEG202 vector (DupLEX-A system, Origene) to obtain the bait plasmids. Bait proteins showing no autonomous activation, and a mouse skeletal muscle cDNA library (DupLEX-A, DLM111, Origene, Rockville, MD, USA) in yeast expression vector pJP4-5, were introduced into yeast strain EGY48 (MATa trp1 his3 ura3 leu2::6 LexAop-LEU2). Transformants were amplified, and positive clones were selected following the protocol recommended in the user manual.

Out of more than 100 positively identified clones, 1 interacting with P1d-8(2α) and P1d-24(2α), but not with P1f-8 or P1f-24, showed 99% identity to a mouse cDNA sequence corresponding to heat shock protein 8/HSC70. Two others, interacting with P1d-24(2α), but not with P1d-8(2α), P1f-8, or P1f-24, showed 99% identity to a mouse cDNA sequence corresponding to α-actinin 3 and synaptopodin 2, respectively.

### 2.8. Co-Immunoprecipitation

Differentiated wild-type myoblasts were scraped off in lysis buffer [20 mM Tris-HCl pH 8.0, 1 mM EDTA pH 8.0, 200 mM NaCl, 0.5% Nonidet P-40, 25 µg/mL PMSF, and cOmplete mini protease inhibitor cocktail (from Roche, Basel, Switzerland)], homogenized by pressing the samples through a 27-gauge needle, centrifuged for 10 min at 10,000× *g* and 4° C, and then the supernatant was used for co-immunoprecipitation. Immunoprecipitation was performed using pan-plectin antibodies and protein A/G sepharose beads (Pierce).

### 2.9. Protein Expression, Purification, and GST-Pull down Assay

For expression of recombinant plectin fragments in bacteria, cDNAs encoding P1d-24(2α) or P1f-24-specific sequences [24] were cloned into the expression vector pKG44 (a modified derivate from pBN120 [25], in which the His-tag was replaced by a GST-tag from pET-42a (Sigma-Aldrich). For bacterial expression of HSC70, the corresponding cDNA was PCR amplified by using primers with EcoRI tails and a mouse skeletal muscle cDNA library (DupLEX-A system, Origene), and cloned into pBN120 (a derivate from pET-15b (Novagene [25])). cDNA encoding human α-actinin 2, a generous gift from K. N. North (Children’s Hospital at Westmead, University of Sydney, Australia), was subcloned into the bacterial expression vector pBN120. Plasmids expressing His-tagged HDAC1, used as a negative control, were kindly provided by C. Seiser (Department of Cell and Developmental Biology, Center for Anatomy and Cell Biology, Medical University of Vienna, Austria).

Fusion proteins were expressed in the *E. coli* strain BL21 (DE3)RIL. GST fusion proteins were purified on gluthathione sepharose 4B beads (GE Healthcare, Chicago, IL, USA), as described in the manufacturer’s instructions. His-tagged fusion proteins were purified using nickel affinity chromatography on His•Bind Resin (Merck, Rahway, NJ, USA) according to the manufacturer’s instructions.

For pull-down experiments, GST-fusion proteins were immobilized on glutathione sepharose 4B beads for 30 min at 4 °C, washed with PBS to remove unbound protein, and incubated with 10–20 µg His-tagged fusion proteins in PBS at 4 °C for 1–4 h with gentle agitation. After centrifugation (500× *g*, 3 min, 4 °C), beads were washed three times with ice-cold wash buffer (20 mM Tris-HCl pH 7.4, 0.1 mM EDTA, 100 mM NaCl, 0.25% Nonidet P-40 for His-tagged α-actinin 2; 20 mM Tris-HCl pH 7.4, 0.1 mM EDTA, 300 mM NaCl, 0.5% Nonidet P-40 for His-tagged HSC70), before being processed for SDS-PAGE and immunoblotting.

### 2.10. Immunoblotting Analyses

Cell and tissue lysates were prepared as described in [16]. Dissected muscles were snap frozen in isopentane cooled with liquid nitrogen, ground in a mortar, and homogenized in lysis buffer (5 mM Tris-HCl pH 6.8, 10% SDS, 0.2 M DTT, 1 mM EDTA, 100 mM NaF, 50 mM ß-glycerophosphate, 2 mM Na_3_VO_4_, 1 mM PMSF, and cOmplete mini protease inhibitor cocktail (Roche)) using a Dounce tissue grinder. The homogenate was centrifuged for 10 min at 10,000× *g*, and the supernatant was mixed with 6× SDS sample buffer (500 mM Tris-HCl pH 6.8, 600 mM DDT, 10% SDS, 0.1% bromophenol blue, 30% glycerol). Cells were directly scraped off in 6× SDS sample buffer and DNA was sheared by pressing the samples through a 27-gauge needle. Samples were boiled at 95 °C for 5 min before being subjected to SDS polyacrylamide gel electrophoresis performed under standard conditions. Proteins were transferred to nitrocellulose membranes (Protran 0.2 NC, Amersham Biosciences, Amersham, UK) using a Mini PROTEAN Tetra Cell blot apparatus (Bio-Rad, Hercules, CA, USA). Membranes were scanned, and the amounts of protein contained in individual bands were quantified using ImageJ software (NIH, Bethesda, MD, USA).

### 2.11. Myoblast Cell Culture

Immortalized skeletal myoblasts were isolated from *Plec*^−/−^ and wild-type littermates, both crossed into a p53^−/−^ background, as described in [13], and cultivated in Ham’s F10 medium (Life Technologies, Carlsbad, CA, USA), supplemented with 20% fetal calf serum (Sigma-Aldrich), 2.5 ng/mL basic fibroblast growth factor (bFGF, Promega, Madison, WI, USA) and antibiotics on collagen-coated (0.01% collagen in PBS, Nutacon, Leimuiden, Netherlands) culture dishes. To induce differentiation, cultures were switched to Dulbecco’s modified Eagle’s medium (Life Technologies), containing 5% horse serum (Life Technologies) and antibiotics.

### 2.12. Cell Stretcher

Stretch experiments were carried out on flexible polydimethylsiloxan (PDMS, Sylgard) substrates coated with laminin-1 (Sigma-Aldrich) that were molded into the shape of a cell culture well with a 2.5 cm^2^ internal surface, as described in [13]. Plated myoblasts were differentiated for 10 days by applying differentiation medium. PDMS gels were then attached to a direct current linear motor with an integrated gearbox (RB35, Conrad Electronic SE, Hirschau, Germany). Uniaxial cyclic stretching was performed in an incubator under normal cell culture conditions for 60 min at 30% stretch amplitude (peak-to-peak) and at a frequency of 0.25 Hz with a resting period (dwell time) of 1 s between the lengthening and the shortening phase. After stretching, cells were homogenized in 6× SDS sample buffer and processed for immunoblotting.

### 2.13. Statistical Analyses

Data analyses and statistical evaluations were performed using Excel 2010 (Microsoft, Redmond, WA, USA) and SPSS Statistics v.19 (IBM, Armonk, NY, USA). Data are given as mean ± SEM. The number of experiments (*N*) and data points (*n*) is indicated in the figure legends. Comparisons between the values of two groups were made using an unpaired, two-tailed Student’s *t*-test (alpha = 0.001–0.05). Comparisons among the values of multiple groups were performed using one-way analysis of variance (ANOVA; alpha = 0.001–0.10). The significance between values of individual groups and controls was subsequently determined using Tukey’s post-hoc test. The *p*-values are * < 0.05, ** < 0.01, and *** < 0.001; a *p*-value < 0.05 was considered statistically significant.

## 3. Results

### 3.1. Plectin Positions Desmin Filaments around Individual Z-Disks

**Figure 1:**
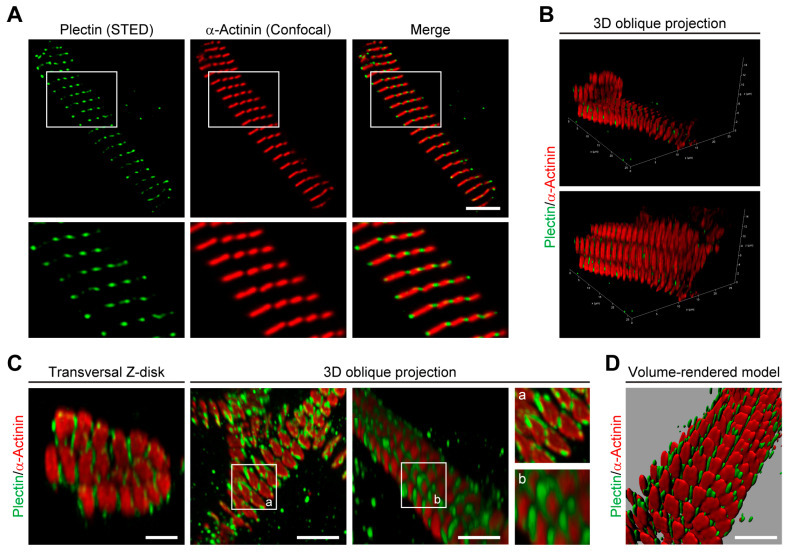
Plectin encases α-actinin-positive Z-disks. (**A**) Combined stimulated emission depletion (STED) imaging and higher-resolution confocal microscopy of myofibril bundles co-immunolabelled using antibodies to plectin and α-actinin. Lower panels are magnifications of boxed areas indicated in upper panels. Note the lateral gaps between the α-actinin-labeled Z-disks of individual myofibrils in the bundle. Additionally, note that plectin is localized in a punctuated pattern interspersing the α-actinin-positive signals. Scale bar: 5 µm. (**B**) 3D images of a myofibril bundle reconstructed from stacks of 2D images in oblique projection and immunolabeled as in (**A**). In the upper panel, a transversal slice in combination with an axial slice of the reconstructed 3D image in the lower panel is shown. Note that in both directions, plectin does not co-localize with the Z-disks but appears in the gaps between individual Z-disks. (**C**) Image deconvolution of isolated individual transversal Z-disk regions and 3D oblique projections indicate that plectin encases α-actinin-positive Z-disks. Insets (a, b) are magnifications of areas showing the spatial arrangement of plectin at the Z-disks. Scale bars: 2 µm. (**D**) Volume-rendered model of myofibrils illustrating plectin’s localization within the gaps between individual α-actinin-positive Z-disks. Scale bar: 5 µm.

Based on immunofluorescence and electron microscopy data, plectin–desmin interaction at the level of Z-disks of striated muscle tissues has been anticipated from early on [26,27,28]. To investigate the spatial arrangement of Z-disk-associated plectin at a resolution below the diffraction limit of conventional confocal microscopy, thin myofibril bundles isolated from mouse psoas and soleus muscles were subjected to STED microscopy after co-immunolabeling for plectin and the Z-disk marker α-actinin. In this way, lateral gaps between α-actinin-labeled Z-disks became apparent. Moreover, a punctuated staining pattern of plectin seemed to intersperse the α-actinin-positive Z-disks and to fill the gaps between them (Figure 1A). As plectin was well aligned within a narrow band of less than 150 nm near the Z-disks, an I-band alignment of plectin could be excluded by the use of high-resolution STED microscopy.

To clarify whether plectin was located inside the bundles, i.e., between individual myofibrils, or just at their periphery, stacks of 2D images of myofibril bundles were obtained. When 3D images were reconstructed from these stacks and viewed in both the transversal and axial direction (Figure 1B), it became obvious that plectin was not localized in the Z-disk interior but in the gaps between the individual Z-disks. This observation was further substantiated by the notion that plectin-positive signals usually border the α-actinin-positive Z-disks, demonstrating that plectin encircles individual myofibrils rather than bundles of multiple myofibrils. In addition, image deconvolution of 3D oblique projections clearly indicated that plectin encases individual α-actinin-positive Z-disks (Figure 1C). Finally, we successfully generated volume-rendered models of skeletal muscle myofibrils (Figure 1D), illustrating that plectin is located within the gap-forming space surrounding the individual α-actinin-positive Z-disks.

**Figure 2:**
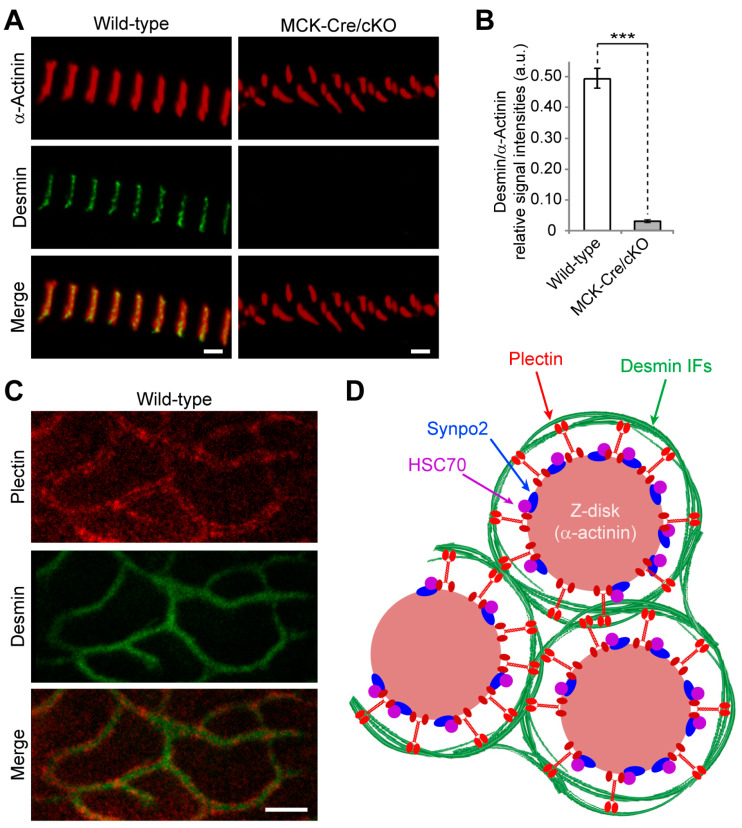
Z-disk-encasing plectin serves as a desmin recruiter. (**A**) Combined STED imaging and higher-resolution confocal microscopy of myofibril bundles isolated from wild-type and conditional, skeletal muscle-specific plectin knockout (MCK-Cre/cKO) mice co-immunolabeled using antibodies to desmin and α-actinin. Note that hardly any desmin signals were obtained at Z-disks of plectin-deficient myofibrils. Scale bars: 2 µm. (**B**) Statistical evaluation of desmin-specific signal at the Z-disk normalized to the α-actinin-specific signal at the same Z-disk. A.u., arbitrary units. Mean ± SEM [wild-type (n = 32), MCK-Cre/cKO (n = 32); *N* = 3]. *** *p* < 0.001, *t*-test. (**C**) Frozen cross-sections of myofibrils were co-immunolabeled using antibodies to plectin and desmin. Note the plectin labeling occurring predominantly between the desmin filaments and the Z-disk. Scale bar: 1 µm. (**D**) Schematic model depicting α-actinin-based Z-disks individually enwrapped by the extrasarcomeric desmin IF network. IFs are connected to and held in place by radially extending plectin (isoform P1d) molecules that interact via their C-terminal (light red endings) IF-binding domains with desmin and via their N-terminal α-actinin-, HCP70-, and synpo2-binding domains (dark red endings) with the Z-disk structure (see text, Section 3.2 and Section 3.3). Note that the model proposes that P1d, by recruiting the IF cytoskeleton selectively to Z-disks, aligns myofibrils laterally and additionally acts as a scaffolding platform for the tension-responsive proteolytic CASA machinery.

In light of plectin’s proposed role as an IF recruiter and Z-disk crosslinking element [4,11], it was of interest to use high-resolution microscopy to also re-evaluate the spatial arrangement of desmin around Z-disks opposite to that of plectin. For this, myofibril bundles isolated from wild-type and striated muscle-specific (MCK-Cre) conditional plectin KO (cKO) mice were analyzed in parallel. While in wild-type myofibril bundles desmin was arranged in a punctuated staining pattern along well-aligned Z-disks and interspersing the α-actinin-positive signals reminiscent of the signals obtained for plectin (Figure 2A, left panels; compare to Figure 1A), almost no desmin-specific staining was observed in myofibrils obtained from MCK-Cre/cKO muscles (Figure 2A, right panels). Furthermore, there were axial shifts in the α-actinin-positive signals, pointing to the loss of structural linkages between Z-disks of neighboring myofibrils in bundles from MCK-Cre/cKO muscles. Statistical quantification of Z-disk-associated desmin-specific signals normalized to α-actinin revealed an absence of desmin at plectin-deficient sarcomeres (Figure 2B).

When frozen cross-sections of myofibril bundles obtained from wild-type soleus muscles were co-immunolabeled for plectin and desmin, double-banded plectin signals between the Z-disks and the extrasarcomeric IF network became apparent (Figure 2C). These observations supported a model where desmin filament bundles, encircling individual Z-disks, are held in place and connected to the α-actinin scaffold of the Z-disk via radially extending plectin molecules (Figure 2D). By recruiting the extrasarcomeric desmin IF cytoskeleton to Z-disks, plectin ensures the lateral alignment of neighboring myofibrils and ascertains the structural integrity of muscle fibers. Together, our data highlight plectin’s crucial role in the organization of the extrasarcomeric IF cytoskeleton.

### 3.2. Loss of Z-Disk-Associated Plectin Leads to Desmin Disconnection, Sarcomere Inhomogeneity, and Altered Relaxation Kinetics of Myofibrils

**Figure 3:**
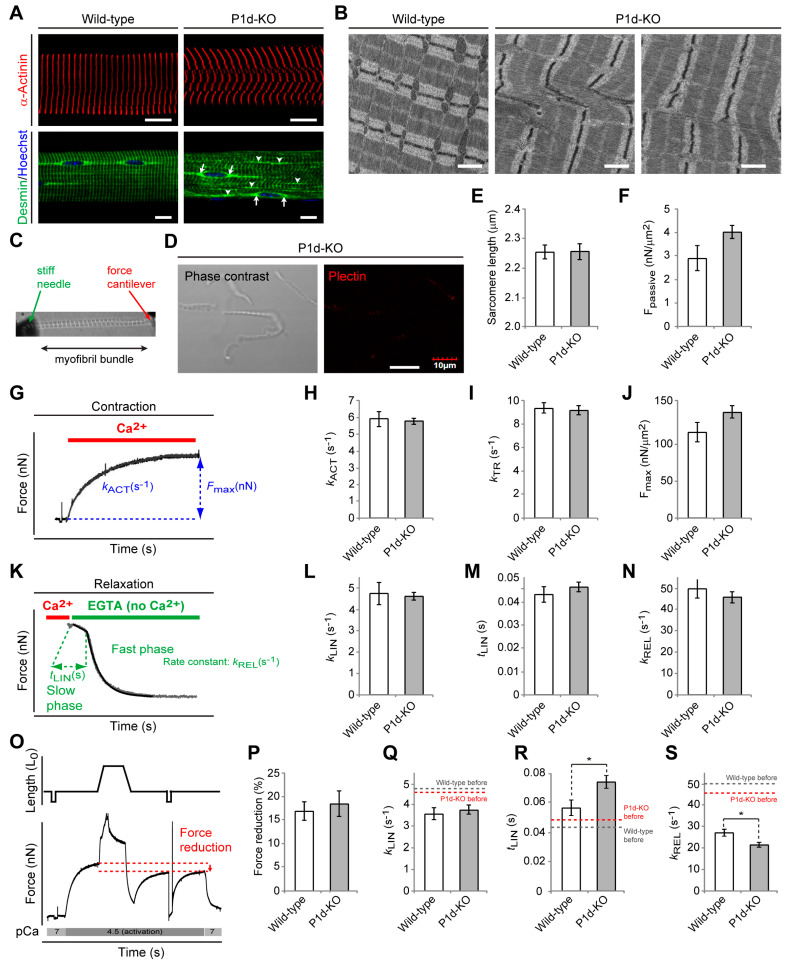
Partial desmin IF collapse, sarcomere inhomogeneity, and altered relaxation kinetics of isoform P1d-deficient myofibrils. (**A**) Immunofluorescence microscopy of teased EDL muscle fibers from wild-type and plectin isoform P1d-deficient (P1d-KO) mice using anti-desmin- (**upper panels**) and anti-α-actinin-specific (**bottom panels**) antibodies. Nuclei are visualized using Hoechst staining in the upper panels. Note the massive accumulation of desmin-positive protein aggregates in the interior of P1d-KO fibers at the level of Z-disks (arrowheads) and the preserved perinuclear desmin staining pattern (arrows). Scale bars: 10 µm. (**B**) Electron micrographs of longitudinal soleus sections showing disturbed Z-band alignment and myofibrillar phase displacement in P1d-KO muscles. Scale bars: 1 µm. (**C**) Picture of a myofibril bundle mounted in the mechanical setup for force measurement. (**D**) Myofibril bundles isolated from P1d-KO mice were immunolabeled using antibodies to plectin. Note that hardly any plectin signals were obtained in P1d-KO myofibrils. Scale bar: 10 µm. (**E**) Statistical evaluation of the sarcomere length of myofibrils isolated from wild-type and P1d-KO mice. Mean ± SEM (wild-type (n = 19), MCK-Cre/cKO (n = 20); *N* = 3). (**F**) Statistical evaluation of the passive tension of wild-type and P1d-KO myofibrils. Mean ± SEM (wild-type (n = 15), MCK-Cre/cKO (n = 21); *N* = 3). (**G**–**J**) Ca^2+^-induced force development of wild-type and P1d-deficient myofibrils was assessed by switching from relaxing solution (pCa 7) to activating solution (pCa 4.5), yielding the maximum Ca^2+^-activated tension F_max_ (shown normalized to cross-sectional area of myofibrils in (**J**)) and the rate constant *k*_ACT_ (**H**). The rate constant *k*_TR_ was derived from the force redevelopment induced by a release–restretch maneuver applied to the bundle during Ca^2+^ activation (**I**). Mean ± SEM ((**H**) wild-type (n = 15), MCK-Cre/cKO (n = 22); (**I**) wild-type (n = 14), MCK-Cre/cKO (n = 21); (**J**) wild-type (n = 15), MCK-Cre/cKO (n = 22); *N* = 3). (**K**) Switching to Ca^2+^-free solution leads to a biphasic relaxation that was fitted by a biphasic function (see Methods section) yielding the rate constant of the slow phase (*k*_LIN_, (**L**)), the duration of the slow phase (*t*_LIN_, (**M**)), and the rate constant of the fast phase (*k*_REL_, (**N**)) of force decay. Mean ± SEM ((**L**–**N**) wild-type (n = 13), MCK-Cre/cKO (n = 19); *N* = 3). (**O**–**S**) Protocol to test mechanical stability of myofibrils against eccentric contractions during Ca^2+^ activation (**O**), statistical evaluations of the force reduction (**P**), the rate constant of the slow phase (*k*_LIN_, (**Q**)), the duration of the slow phase (*t*_LIN_, (**R**)), and the rate constant of the fast phase (*k*_REL_, (**S**)) after eccentric contraction. Note that, in general, relaxation kinetics were slower after the eccentric contraction protocol in myofibril bundles from wild-type and P1d-KO mice (dashed lines in (**Q**–**S**) represent values of respective parameters before the eccentric protocol, as shown in (**L**–**N**)). Also note that P1d deficiency enhances this effect, as the duration of the slow phase was significantly prolonged and the rate constant of the fast phase was significantly reduced after eccentric exercise in P1d-KO compared to wild-type myofibrils. Mean ± SEM ((**P**) wild-type (n = 15), MCK-Cre/cKO (n = 21); (**Q**–**S**) wild-type (n = 13), MCK-Cre/cKO (n = 19); *N* = 3). * *p* < 0.05 compared with wild-type, *t*-test.

Out of the multiple plectin isoforms expressed in skeletal muscle tissue, only one, isoform P1d, shows specific association with Z-disks [11,12,13]. Hence, plectin isoform 1d-specific knockout (P1d-KO) mice [11], specifically lacking this isoform while expressing all others, enabled us to study the role of Z-disk-associated plectin in more detail. As sarcomere disruption and accumulation of desmin-positive protein aggregates are the major hallmarks of myofiber degeneration in plectin-related myofibrillar myopathies [3,13], we evaluated Z-disk appearance and the IF network distribution in teased extensor digitorum longus (EDL) fibers isolated from wild-type and P1d-KO mice using immunofluorescence microscopy for α-actinin or desmin, respectively (Figure 3A; see also [11]). While highly organized cross-striated staining patterns were visualized in wild-type fibers, P1d-KO fibers manifested with disorganized myofibrillar structures, displaying a disoriented rather than continuous transverse α-actinin-specific staining pattern (Figure 3A, upper panels). Moreover, desmin was localized in perinuclear areas and along regularly organized sarcomeres in wild-type myofibers, while in the P1d-mutant fibers, desmin-positive Z-disks appeared misaligned, and areas with aggregated desmin IFs became apparent (Figure 3A, bottom panels, arrowheads), confirming that the desmin IF network is partially detached from the Z-disks and accumulates in their vicinity. Interestingly, the perinuclear desmin staining was preserved in P1d-KO fibers (Figure 3A, arrows), indicating that the lack of P1d did not affect the desmin IF network at the nuclear periphery, contrary to the lack of isoform P1 [29] (Figure 3A, bottom panels). Together with previous observations [11], these data reassure the isoform-specific role of P1d in preserving the regular alignment of sarcomeric structures and enabling a functional desmin IF network. Extending these observations to the ultrastructural level, we subjected longitudinal sections of P1d-KO soleus muscle to transmission electron microscopy (Figure 3B). In this way, lateral shifts and distortions of adjacent sarcomeric units leading to disturbed Z-band alignment and myofibrillar phase displacement could be clearly visualized in P1d-deficient specimens. Misalignments of this type were hardly ever found in corresponding wild-type specimens.

To investigate whether the P1d deficiency-inflicted alterations in myofibril alignment affected functional aspects, we subjected thin myofibril bundle preparations derived from P1d-KO and wild-type psoas muscles to biomechanical measurements. For this purpose, thin myofibril bundles were mounted between a stiff needle and an atomic force cantilever in an apparatus that enabled rapid activation and relaxation of the myofibrils by a microflow solution exchange technique (Figure 3C; [21]). Because thin myofibril bundles are not diffusion limited, they are ideal for kinetic and biomechanical analysis of sarcomere function. In initial control experiments, the lack of Z-disk-associated plectin in P1d-KO psoas myofibrils was confirmed by negative results in immunoblotting with antibodies to plectin that did not discriminate between isoforms (anti-pan-plectin) and consequently would have detected any isoform substituting for P1d (Figure 3D). We also showed that within a bundle, the overall sarcomere lengths of myofibrils isolated from P1d-KO mice were comparable to those from wild-type animals, indicating that the contractile units per se were not altered (Figure 3E). Moreover, upon stretching under relaxing conditions to the same sarcomere length of 2.45 µm, P1d-KO and wild-type myofibrils revealed a similar passive tension (Figure 3F). When the Ca^2+^-dependent contractility of P1d-KO compared to wild-type myofibrils was examined (Figure 3G), the rate constants of Ca^2+^-induced force development (*k*_ACT_, Figure 3H), mechanically-induced force re-development (*k*_TR_, Figure 3), as well as the maximum Ca^2+^-activated tension (F_max_, Figure 3J), were comparable for both types of myofibrils, indicating that myofibrils from P1d-KO mice showed no alterations in cross-bridge turnover kinetics and active force development.

Next, by rapidly reducing the calcium concentration, the kinetics of the biphasic force relaxation were assessed (Figure 3K). Interestingly, neither the deduced rate constant of the slow phase (kLIN, Figure 3L), nor the duration of the slow phase (tLIN, Figure 2M), nor the rate constant of the fast phase (kREL, Figure 3N), differed between P1d-KO and wild-type samples. Finally, to test whether myofibril bundles lacking plectin are more vulnerable to mechanical stress, they were stretched during active contraction (Figure 3O). Even such eccentric contractions resulted in a similar loss of force in wild-type and P1d-deficient myofibril bundles (Figure 3P). During the course of these experiments, the relaxation kinetics of myofibril bundles from both wild-type and P1d-KO muscles became slower after the eccentric contraction protocol compared to the initial measurements, as denoted by the reduced rate constants of the slow phase and fast phase, while the duration of the slow phase of relaxation was increased (Figure 3Q–S; dashed lines indicate respective evaluations for kLIN, tLIN, and kREL before the eccentric protocol). Strikingly, however, this slow-down effect was clearly enhanced by the lack of plectin, as the duration of the slow phase was significantly prolonged and the rate constant of the fast phase was significantly reduced after eccentric exercise in myofibrils from P1d-KO compared to wild-type muscles. This indicated a manifestation of altered relaxation kinetics upon mechanical stress. Moreover, as relaxation kinetics are determined by well-coordinated sarcomere dynamics, these results are in line with the observed sarcomere inhomogeneity of P1d-deficient skeletal muscle fibers, supporting its physiological significance.

### 3.3. Specific Interaction of P1d with the Z-Disk-Associated Proteins α-Actinin, HSC70, and Synpo2

**Figure 4:**
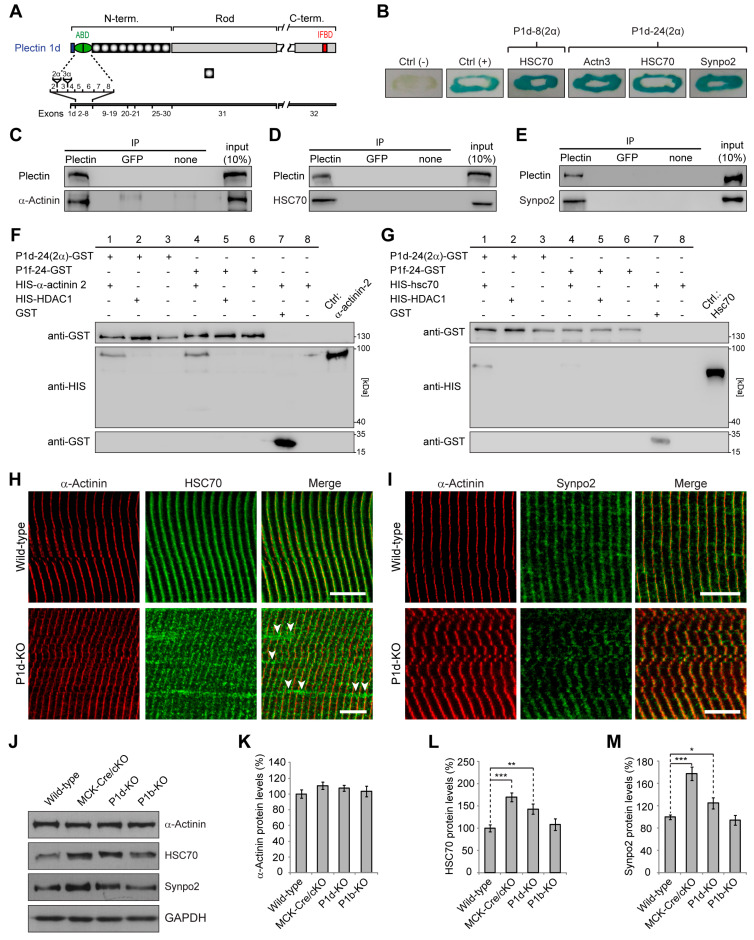
Specific interaction of plectin isoform P1d with the Z-disk-associated proteins HSC70, α-actinin, and synpo2. (**A**) Schematic representation of plectin subdomains and exon allocation. Abbreviations are as follows: N- and C-term., N- and C-terminal domains; ABD, actin binding domain; IFDB, intermediate filament binding domain. (**B**) Verification of novel P1d-specific interaction partners identified in a yeast two-hybrid screening (using plectin fragments P1d-8(2α) and P1d-24(2α) as bait) with a β-galactosidase assay. Blue colonies indicate positive interaction between the bait and the respective protein. Note that only HSC70 interacts with the shorter P1d fragment (P1d-8(2α)), while α-actinin, HSC70, and synpo2 were identified when the longer P1d fragment (P1d-24(2α)) was used as bait. (**C**–**E**) Co-immunoprecipitation of plectin and α-actinin (**C**), HSC70 (**D**), or synpo2 (**E**). Myoblast cell lysates were subjected to immunoprecipitation using pan-plectin antibodies, or antibodies to GFP (as control), or A/G sepharose beads alone (none), and precipitated proteins were detected by immunoblotting using antibodies to proteins as indicated. (**F**) In vitro pull-down of His-tagged α-actinin by GST-tagged P1d-24(2α) and P1f-24. HIS-HDAC1 and GST were used as negative controls. (**G**) In vitro pull-down of His-tagged HSC70 by GST-tagged P1d-24(2α), but not by GST-tagged P1f-24, revealed that the binding to HSC70 was isoform P1d-specific. HIS-HDAC1 and GST were used as negative controls. (**H**) Immunofluorescence microscopy of wild-type and P1d-KO teased muscle fibers using antibodies specific for α-actinin and HSC70. Note that HSC70 co-localizes with α-actinin in both wild-type and P1d-KO muscle fibers. In addition, there are HSC70-positive areas not co-localizing with α-actinin in P1d-KO fibers (arrowheads). Scale bars: 10 µm. (**I**) Immunofluorescence microscopy of wild-type and P1d-KO teased muscle fibers using antibodies specific for α-actinin and synpo2. Note that synpo2 co-localizes with α-actinin in wild-type and P1d-KO muscle fibers. Scale bars: 10 µm. (**J**) Equal amounts of wild-type, MCK-Cre/cKO, P1d-KO, and P1b-KO lower leg muscle lysates were subjected to immunoblotting using antibodies as indicated. GAPDH, loading control. (**K**–**M**) Signal intensities of immunoblots as shown in (**J**) were densitometrically measured and normalized to total protein content, as analyzed by Coomassie staining (not shown). Mean ± SEM; *N* = 6–8. * *p* > 0.05, ** *p* > 0.01, *** *p* < 0.001, compared to wild-type, *t*-test. Note increased expression levels of HSC70 and synpo2, but not of α-actinin, in MCK-Cre/cKO and P1d-KO muscle lysates compared to wild-type and P1b-KO samples. Original immunoblots see Figures S1–S6.

To explore targeting mechanism and specific functions of P1d, we screened for P1d-specific binding partners using yeast two-hybrid screening with a mouse skeletal muscle cDNA library and plectin isoform-specific baits. To identify binding partners interacting with the isoform-specific N-terminal domain of P1d, two different bait proteins were used. One of them, encoded by exons 1d–8, represented the N-terminal, a five amino acid residue-long, isoform P1d-specific sequence and the ensuing actin-binding domain (ABD), while the other (encoded by exons 1d–24) included most of plectin’s plakin domain (Figure 4A). Corresponding sequences of plectin isoform 1f (P1f) were used as controls. Moreover, since it has been shown that the sequence of muscle-specific P1d contains within its ABD five additional amino acid residues that are encoded by an alternatively spliced exon 2α (Figure 4A, [24]), the corresponding 2α sequence was included in both P1d baits, termed as P1d-8(2α) and P1d-24(2α), respectively. This type of screening led to the identification of a number of proteins that were binding specifically to either P1d or P1f. Prominent among the specific P1d-binding partners were the muscle-specific Z-disk component α-actinin 3, heat shock cognate protein 70 (HSC70; also known as HSPA8), and synaptopodin 2 (synpo2; also known as myopodin) (Figure 4B). Of these, only HSC70 was found to interact with the shorter P1d variant P1d-8(2α), while all three of these proteins were identified when the longer P1d fragment, P1d-24(2α), was used as bait (Figure 4B). Alpha-actinin, HSC70, and synpo2, could all be co-immunoprecipitated with plectin from lysates of an immortalized (p53-deficient) plectin wild-type (*Plec^+/+^*) myoblast cell line [13] (Figure 4C–E), verifying their tight association with plectin. To further validate the binding specificity of muscle-specific α-actinin and HSC70 for plectin isoform P1d, in vitro pull-down assays were performed, where His-tagged versions of α-actinin isoforms 2 and 3, or HSC70, were used as prey, and GST-tagged P1d-24(2α) or GST-tagged P1f-24 were used as bait (Figure 4F,G; data not shown). It was found that both plectin isoform versions, P1d-24(2α) and P1f-24, were pulling down and, thus, directly interacted with muscle-specific α-actinin (Figure 4F), opposite to HSC70 which interacted only with P1d-24(2α) but not P1f-24 (Figure 4G). This indicated that only HSC70 bound to plectin in an isoform-(P1d)-specific manner. Moreover, these data supported the notion that P1d’s interaction with HSC70 was important for its specific targeting to the Z-disk.

Alpha-actinin is well established as the major component of Z-disks [30], while HSC70 as well as synpo2 have been identified as Z-disk-associated proteins [31,32] and as components of the CASA machinery, which is activated upon tension and is essential for muscle maintenance [33,34,35,36]. To assess the effects of P1d deficiency on the subcellular localization of either HSC70 and synpo2, teased EDL muscle fibers isolated from wild-type and P1d-KO mice were subjected to double immunofluorescence microscopy for α-actinin with either HSC70 or synpo2 (Figure 4H,I). While in wild-type muscle fibers, α-actinin-positive Z-disks appeared in a regular striated pattern, in P1d-KO myofibers they were visualized as misaligned and in part disoriented structures (Figure 4H). In both wild-type and P1d-KO fibers, HSC70 co-localized with the Z-disk structures, but, interestingly, additional HSC70-positive areas, which were not co-localizing with α-actinin, were detected along P1d-deficient (Figure 4H, arrowheads), but not wild-type, muscle fibers. The double immunolabeling of wild-type and P1d-deficient muscle fibers for synpo2/α-actinin revealed exclusively co-localization of both proteins at the level of Z-disks, with no signals detectable in Z-disk-remote areas (Figure 4I).

Moreover, to evaluate the expression levels of α-actinin, HSC70, and synpo2 in P1d-deficient tissue, lower leg muscle lysates were prepared from wild-type and P1d-KO mice, and were analyzed by immunoblotting. To assess whether any observed changes were P1d-specific, we included in this analysis muscle lysates from MCK-Cre/cKO mice (devoid of all plectin isoforms) and from mice lacking plectin isoform P1b (P1b-KO [14]) instead of P1d (Figure 4J). The statistical evaluation of signal intensities of immunoblots showed that the protein levels of HSC70 and synpo2 were significantly increased to ~170% and ~145%, and to ~180% and ~125% in MCK-Cre/cKO and in P1d-KO muscles, respectively, compared to wild-type muscle. In contrast, the different muscle lysates showed no differences in α-actinin expression. In P1b-deficient muscle lysates, none of the proteins tested showed changes in expression levels compared to wild-type, indicating that the observed effects were indeed isoform P1d-specific (Figure 4K–M). Taken together, these experiments confirmed that P1d is anchored at the Z-disk via direct interaction with α-actinin and, additionally, that it interacts with the CASA machinery via specific binding to HSC70 and synpo2. However, the Z-disk-specific localization of HSC70 and synpo2 seemed to be independent of P1d expression, as both proteins were still aligned with Z-disks in P1d-KO myofibers. A schematic presentation of plectin’s Z-disk-associated interaction partners has been included in Figure 2D.

### 3.4. P1d Deficiency Leads to Increased Levels of CASA Constituents as Well as the Substrate Protein Filamin C

**Figure 5:**
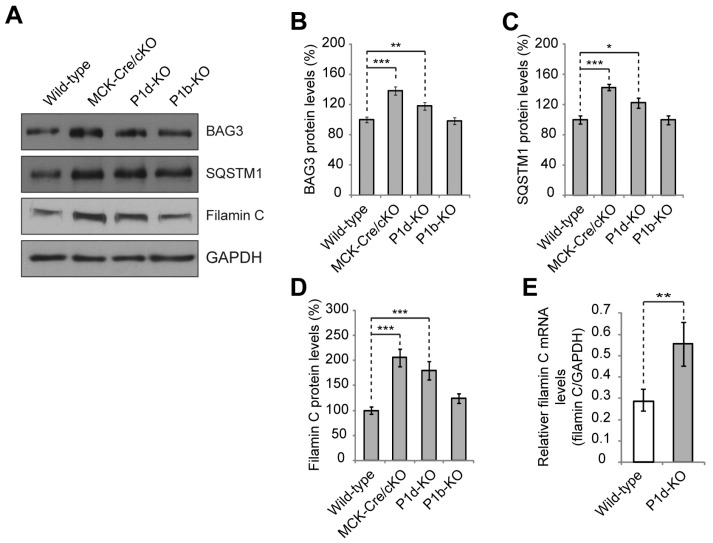
Increased levels of CASA-associated proteins BAG3, SQSTM1, and filamin C in P1d-deficent muscle. (**A**) Equal amounts of wild-type, MCK-Cre/cKO, P1d-KO, and P1b-KO lower leg muscle lysates were subjected to immunoblotting using antibodies as indicated. GAPDH, loading control. (**B**–**D**) Signal intensities of immunoblots as shown in (**A**) were densitometrically measured and normalized to total protein content, as analyzed by Coomassie staining (not shown). Mean ± SEM; *N* = 6–8. * *p* > 0.05, ** *p* > 0.01, *** *p*< 0.001, compared to wild-type, *t*-test. Note the increased expression levels of CASA-associated proteins in MCK-Cre/cKO and P1d-KO muscle lysates compared to wild-type and P1b-KO samples. (**E**) Statistical evaluation of filamin C mRNA levels in gastrocnemius muscle of wild-type and P1d-KO mice. Original immunoblots see Figure S7.

It has been shown that the CASA machinery senses mechanical stress of the muscle fiber and leads to degradation of large cytoskeletal components damaged during contraction [33,34,35,36]. During this process, contraction of the actin network leads to the unfolding of the actin-crosslinking and -anchoring protein filamin C and initiates the autophagic disposal of mechanically damaged copies of the protein. HSC70, together with the co-chaperone HSP22, is essential for the initiation of CASA, while the autophagosome formation during CASA is dependent on an interaction of BAG3 with synpo2. Finally, p62/SQSTM1 acts as an autophagic receptor for the CASA complexes necessary for engulfing cargoes into membranous phagophore structures [34,35,36]. Because HSC70 and synpo2 protein levels were found to be increased in P1d-KO muscle lysates, we questioned whether other components of the CASA machinery, such as BAG3 or SQSTM1, as well as specific CASA-substrates, such as filamin C, displayed altered expression in P1d deficiency as well. For this purpose, skeletal muscle lysates derived from wild-type, MCK-Cre/cKO, P1d-KO, and P1b-KO were analyzed by immunoblotting (Figure 5A). Statistical evaluations of signal intensities of immunoblots revealed that the protein levels of BAG3 and SQSTM1 were significantly increased to ~140% and ~120%, and to ~145% and ~125% in MCK-Cre/cKO and P1d-KO muscles, respectively, compared to wild-type samples, while no alterations in expression levels were observed for P1b-KO lysates (Figure 5B,C). Moreover, the expression levels of the bona fide CASA substrate filamin C were found to be substantially increased (to ~200% and ~180%), in MCK-Cre/cKO and P1d-KO muscles, respectively, compared to wild-type levels (Figure 5D). The insignificance of a corresponding phenotype in P1b-KO muscle again underlined isoform (P1d) specificity. To obtain an indication of whether increased filamin C protein levels were due to increased expression or impaired degradation, we also measured filamin C mRNA levels comparatively in wild-type and P1d-KO skeletal muscle samples. We found filamin C mRNA expression in P1d-deficient muscle to be upregulated by a factor of 1.8 (Figure 5E), which is comparable to the increase observed in the protein level (compared to Figure 5D). However, it remains to be investigated why the sarcomeric protein quality control machinery, in spite of being upregulated, did not lead to reduced filamin C levels and, thus, was functionally impaired in the case of P1d deficiency. Ineffectiveness of the CASA machinery, including inaccessibility or dislocation of its substrates, could be the reason for the accumulation and eventual aggregation of misfolded proteins in the affected muscles.

### 3.5. Mechanical Strain Triggers Excessive Elevation of Filamin C Levels in Plectin-Deficient Myotubes

**Figure 6:**
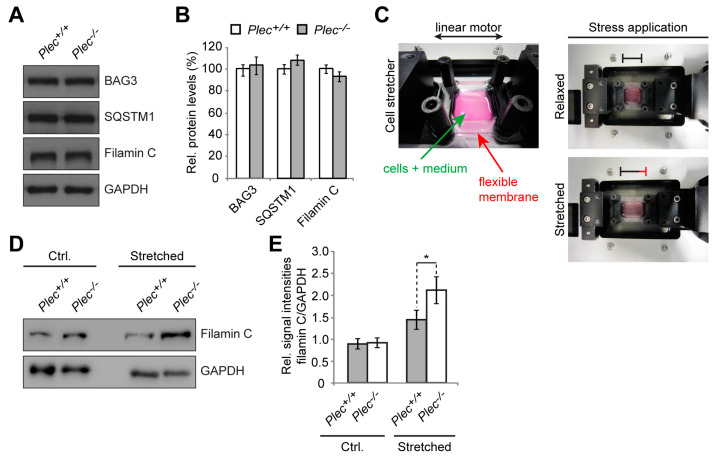
Upregulation of filamin C in plectin-deficient myotubes after mechanical stretching. (**A**) Cell lysates prepared from differentiated wild-type (*Plec^+/+^*) and plectin-deficient (*Plec*^−/−^) myoblasts were subjected to immunoblotting using antibodies as indicated. Note that the expression of CASA-associated proteins was not altered in plectin-deficient cells. GAPDH, loading control. (**B**) Signal intensities of CASA-associated protein bands as shown in (**A**) were densitometrically measured and normalized to the total protein content, as assessed by Coomassie staining (not shown). Mean ± SEM; *N* = 3. (**C**) Photographic image of the cell stretcher device used in this study. Myoblasts were differentiated for 10 days on a flexible PDMS-membrane and, to apply mechanical strain, the membrane was stretched up to 30% by a linear motor. (**D**) Quantitative immunoblotting analysis of cell lysates derived from untreated and mechanically stretched *Plec^+/+^* and *Plec*^−/−^ myotubes using antibodies specific for filamin C. GAPDH was used as the loading control. (**E**) Statistical evaluation of signal intensities of filamin C protein bands as shown in (**D**) normalized to the total protein content (GAPDH signal intensities). Note the drastic increase in filamin C levels in stretched *Plec*^−/−^ myotubes compared to the moderate filamin C upregulation in *Plec^+/+^* cells. Mean ± SEM; *N* = 3. * *p* > 0.05, *t*-test. Original immunoblots see Figures S8 and S9.

As CASA was shown to be a tension-induced machinery activated upon high loads of mechanical exercise and muscle contraction [33,34,35,36,37], and in light of P1d-deficient myofibril bundles displaying altered relaxation kinetics upon mechanical stress (see Figure 3Q–S), we wanted to establish a monitoring system for measuring the impact of mechanical strain on plectin (1d)-deficient myofibrils in an ex vivo cell culture system. In previous studies, we showed that plectin-deficient (*Plec*^−/−^) myoblasts can be differentiated ex vivo into multinucleated myotubes which closely mirrored the pathology of plectin-deficient muscle fibers, including desmin-positive protein aggregates and Z-disk aberrations, thereby representing a reliable tool for investigations at the cellular level [13]. Moreover, *Plec*^−/−^ myotubes were more susceptible to mechanical strain, as shown by increased detachment from a flexible membrane compared to *Plec^+/+^* cells when exposed to cyclic stretch using a cell stretcher [13]. When we performed immunoblotting experiments of cell lysates prepared from differentiated *Plec^+/+^* and *Plec*^−/−^ myotubes using antibodies to BAG3, SQSTM1, and filamin C (Figure 6A), no changes in expression were observed for any of the proteins evaluated (Figure 6B). This was not unexpected, considering that contrary to myofibers from muscle tissues, ex vivo formed myotubes in culture are not fully differentiated and are hardly exposed to mechanical strain. We then attempted to induce the CASA machinery in cultured myotubes by subjecting the cells to a mechanical strain mimicking muscle contraction [38,39], by cultivating myoblasts on the flexible membrane of a cell stretching device, differentiating them into myotubes, and subsequently subjecting them to mechanical stress by continuous stretching and relaxing of the membrane (Figure 6C). Using filamin C as representative readout for CASA activity upon tension-induced damage, we found that its protein levels were indeed increased upon stretching in both types of myotubes (Figure 6D,E). Interestingly, however, in *Plec*^−/−^ myotubes, the increase in filamin C expression was ~50% higher than in their *Plec*^+/+^ counterparts (Figure 6E), indicating the rather drastic impact of plectin deficiency. Again, an impaired activity of the CASA machinery in myocytes lacking plectin could be a possible mechanistic explanation for this phenotype. In any case, the data set obtained with ex vivo differentiated *Plec*^−/−^ myotubes together with the one from P1d-KO tissue-derived myofibrils, implied that plectin-deficient, in particular isoform P1d-deficient, muscle cells were more susceptible to damage upon exposure to mechanical strain.

### 3.6. Physical Exercise of P1d-Deficient Mice Leads to Their Early Exhaustion, Filamin C Protein Aggregation, and Development of Myofibrillar Lesions

**Figure 7:**
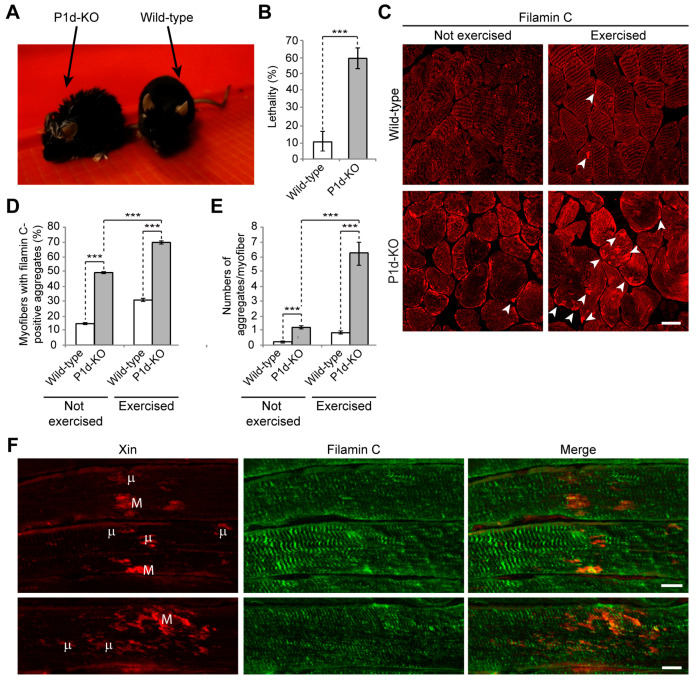
Forced swim experiments with P1d-KO mice reveal early exhaustion, increased lethality, and filamin C-positive sarcomeric lesions in muscle tissue. (**A**) Photographic images of wild-type and P1d-KO mice after swimming on the last training day. Note that P1d-KO mice, contrary to their wild-type littermates, were unable to clean their fur immediately after the swim experiment, because they were too exhausted and had to rest. (**B**) Statistical evaluation of mice which died during the swim experiment. Note the drastic lethality of P1d-KO mice. Mean ± SEM [wild-type (n = 27), P1d-KO (n = 30)]. *** *p*< 0.001, *t*-test. (**C**) Immunofluorescence microscopy of skeletal muscle sections (soleus) derived from wild-type and P1d-KO mice, either left untreated or exercised during forced swim experiments. Note the increased appearance of filamin C-positive protein aggregates (arrowheads) in P1d-KO mice after the swim experiment. Scale bar: 10 µm. (**D**) Statistical evaluation of the number of myofibers containing filamin C-positive protein aggregates. (**E**) Statistical evaluation of the number of filamin C-positive protein aggregates per individual myofiber. (**D**,**E**) Only myofibers containing at least 1 filamin C-positive protein aggregate with a size ≥ 3 µm were taken into consideration. Mean ± SEM [muscle fibers analyzed: wild-type, not exercised (n = 209), P1d-KO, not exercised (n = 215), wild-type, exercised (n = 286), P1d-KO, exercised (n = 141); *N* = 3]. *** *p*< 0.001, *t*-test. (**F**) Double immunofluorescence microscopy of longitudinal soleus muscle sections derived from P1d-KO mice after the forced swim test using antibodies specific for Xin and filamin C. Note the presence of filamin C- and Xin-double-labeled microlesions (µ, spanning 1–5 sarcomeres) and macrolesions (M, spanning areas larger than 5 sarcomeres). Scale bars: 10 µm.

As CASA is a tension-induced degradation pathway [34,35,36,37], it was of prime interest to assess the impact of P1d deficiency on muscle integrity on the organismal level. For this reason, wild-type and P1d-KO mice were subjected to a forced swim test involving intense muscle training. During this endurance test, mice were trained for 2 weeks in two daily swim sessions that lasted 20 min at the start and were increased by 10 min every day until 90 min of swimming were reached. In general, P1d-KO mice became exhausted much faster than wild-type mice, so frequently they had to be removed from the water and were allowed to rest before resuming the session. When removed from the water, P1d-KO mice were often too exhausted to clean their fur, as is usually carried out by wild-type mice immediately after swimming; mutant mice also trembled and breathed heavily (Figure 7A). Ultimately, almost 60% of P1d-KO mice died during the swimming experiment, probably due to heart failure (Figure 7B).

Skeletal muscle cryosections of wild-type mice showed a regular filamin C staining pattern underneath the sarcolemma and at the level of Z-disks, regardless of whether the mice were trained or not (Figure 7C, upper panels); sporadically, filamin C-positive aggregates (arrowheads) became apparent after swimming. However, in P1d-deficient muscle (Figure 7C, lower panels) the situation was different, as in untrained as well as in trained P1d-KO mice, the endogenous filamin C pattern appeared largely disrupted, with occasional subsarcolemmal filamin C-positive protein aggregates being visible already prior to training (Figure 7C, lower left panel, arrowhead), and a drastic increase in the number of such aggregates after swimming (Figure 7C, lower right panel, arrowheads). Statistical analyses revealed that almost 50% of untrained P1d-KO muscle fibers contained filamin C-positive aggregates with a diameter of >3 μm, while only 15% of wild-type counterparts displayed aggregates of such size. Upon swimming, the percentage of myofibers containing filamin C-positive aggregates drastically increased to 70% in trained P1d-KO mice, compared to a moderate increase to 30% in wild-type mice (Figure 7D). Moreover, the number of filamin C-positive aggregates (>3 μm) per myofiber increased from ~1.5 aggregates in untrained P1d-KO mice to ~7 aggregates in trained P1d-KO animals (Figure 7E).

Eccentric exercise leads to focal disruptions in myofibrils, referred to as “lesions”, which are morphological hallmarks of contraction-induced injury [40]. To assess myofibrillar instability in exercised muscles of P1d-KO mice by the presence of such lesions, longitudinal sections of soleus muscle prepared from mice shortly after swimming were immunolabeled for Xin, a known marker for sarcomeric lesions [40]. Double immunofluorescence microscopy, using antibodies to Xin and filamin C combined, revealed the formation of Xin-positive micro- and macro-lesions, situated at the periphery of myofibrils and spanning over 1–5, or more than 5 sarcomeres, respectively (Figure 7F). A similar phenotype has been described for human and murine filaminopathies [41], highlighting the detrimental repercussions of P1d deficiency on skeletal muscle integrity.

## 4. Discussion

Investigating the spatial arrangement of plectin molecules at the Z-disks of myofibers by super-resolution immunofluorescence microscopy, we show here that plectin is localized in the α-actinin-negative gaps existing between the laterally aligned Z-disks but is missing from the interior of Z-disks. This notion was substantiated by the observation of plectin-positive signals bordering, but not overlapping with, the α-actinin-positive Z-disks. Our data further demonstrate that plectin-positive structures encircle individual (single) myofibrils rather than bundles of multiple myofibrils, whereby the encasing of individual α-actinin-positive Z-disks by plectin molecules became clearly evident by image deconvolution of 3D oblique projections (Figure 1C). Finally, we could successfully generate volume-rendered models of skeletal muscle myofibrils, illustrating that plectin is located within the gaps between individual α-actinin-positive Z-disks (Figure 1D). These experiments add to and complement a previous immunogold electron microscopy study, in which fine plectin threads were found to be located between Z-disks and IF networks in skeletal muscle sections [28]. STED microscopy of transversal muscle cryosections provides direct structural evidence for previous models [11,28], suggesting that plectin is located between Z-disks and IFs. Our results are in line with the concept of plectin acting as a universal IF recruiting and anchoring platform. The lack of any desmin-specific staining at the Z-disks of plectin-deficient myofibril bundles confirmed that the association of desmin with Z-disks was plectin-dependent and desmin was dissociated from myofibrillar Z-disks in the absence of plectin linkages. Dispatched desmin filaments consequently collapse and accumulate between plectin-deficient myofibril bundles, thereby generating the desmin-positive protein aggregates observed in EBS-MD patients, MCK-Cre/cKO mice, and plectin-deficient myotubes [11,13,42]. Similar to plectin, missense mutations in desmin have been shown to compromise the structure and maintenance of IF filaments on various levels, frequently causing abnormal cytoplasmic desmin aggregates [43,44]. However, while desmin-related myopathies and cardiomyopathies are mostly caused by filament formation defects, in plectinopathies, desmin aggregation is a consequence of missing linkages between the IFs and other structures, such as the Z-disks.

Plectin’s versatility is largely based on the differential cellular targeting of its individual isoforms. As we show here, microscopic and ultrastructural analyses revealed that the loss of Z-disk-associated P1d leads to highly disorganized myofibrillar structures and phase displacement of sarcomeric structures, pointing towards increased inhomogeneity of the muscle structure. Detachment and loss of regularly anchored desmin IFs, as observed in teased muscle fibers from P1d-KO mice, led to the formation of desmin aggregates in interior areas of the fiber. Moreover, peripheral and perinuclear desmin-positive signals indicated that other plectin isoforms expressed in muscle fibers, such as P1f and P1, were still intact. On the other hand, in plectin-stained myofibril bundles from P1d-KO mice, no remaining plectin signals were observed, reassuring us that P1d is the sole Z-disk-associated plectin isoform. In line with previous observations [11,13] our analyses emphasize the unique role of isoform P1d in maintaining regular myofibril alignment. The striking sarcomere pathology, as observed in muscle from EBS-MD patients, could, therefore, be attributed primarily to a loss of function of isoform P1d. When we assessed the mechanical performance of myofibrillar bundles, P1d-deficient myofibrils displayed similar passive tension, comparable Ca^2+^-dependent contraction abilities, and unaltered biphasic force relaxation compared to wild-type samples. However, when we tested the vulnerability to mechanical stress by eccentric contractions, despite displaying a similar force reduction to wild-type myofibrils, myofibril bundles from P1d-KO revealed a significantly prolonged duration of the slow phase and reduced rate constant of the fast phase after the eccentric contraction. In the context of biomechanical studies that indicate organized inter-sarcomere dynamics as the cause of rapid relaxation [21,45,46], our results suggest that this mechanism of relaxation is impaired upon mechanical stress, particularly when P1d is not available for stabilizing the inter-myofibrillar sarcomere organization.

Previous conventional confocal immunofluorescence microscopy studies on myofibers revealed that of the four major plectin isoforms expressed in mature cells, i.e., P1, P1b, P1d, and P1f, only P1d co-localized with desmin at the level of the Z-disks, while P1f was localized at the periphery of cells along the sarcolemma, P1 in perinuclear areas, and P1b in association with mitochondria. In previous studies, we have identified isoform-specific binding partners that show co-localization with isoforms at their targeting sites, in the cases of P1b, P1f, and P1 [12,14,29], strongly suggesting that isoform-specific interactions are a basic part of the mechanisms underlying isoform-, and, by implication, IF network-targeting. The mechanism of P1d-mediated docking of desmin IFs at a sarcomeric Z-disks-associated platform remained unresolved. The existence of such a platform was implicit, however, based on similar sarcomeric phenotype manifestations of isoform P1d-deficient and desmin-null mice, such as disarrayed disks paired with misaligned myofibrils [11,13,47]. Using Y2H screening combined with co-immunoprecipitation and in vitro pull-down assays, we here identified several Z-disk-associated proteins as direct interaction partners of P1d, among them the bona fide Z-disk constituent protein α-actinin. However, plectin’s binding to α-actinin was found to be isoform-independent, as it also occurred with N-terminal fragments of plectin corresponding to isoforms other than P1d (see Figure 4F). In fact, there is evidence from previous studies that plectin’s α-actinin-binding domain resides within plectin’s (spectrin repeats-containing) plakin domain that resides downstream of the ABD and precedes the central α-helical rod domain (unpublished data from the authors’ laboratory). Thus, the binding affinity to α-actinin, as important as it might be for P1d’s Z-disk-related functions, is unlikely to be the driving force for its recruitment to Z-disks. On the other hand, our experiments show that the N-terminal fragment of P1d binds in an isoform-specific manner to HSC70, a constitutively expressed chaperone protein involved in diverse cellular processes including protein folding and protein degradation. In mature myofibers, HSC70 is found predominantly at the level of Z-disks (Figure 4H) and, as we show here, its association with Z-disks is independent of P1d. Thus, HSC70 qualifies as an isoform-specific binding protein that might be instrumental in recruiting P1d, and thereby the desmin network, to Z-disks of the contractile apparatus.

Whether only the five amino acid residue-long isoform-specific protein sequence preceding P1d’s ABD suffices for specific HSC70-binding, or whether the first exon-encoded protein domain acts in combination with the succeeding ABD, remains to be determined. Targeting signals or site-specific interaction domains residing in isoform-specific head domains have been described for a number of isoforms [10], but only in the case of P1b was the head domain shown to suffice for site-specific targeting [10,14]. In most other cases, the experiments reported point towards an involvement and combined action of the specific head domains with the adjacent ABD in determining targeting preferences. To understand the isoform-dependent plasticity of plectin’s ABD and the conformational changes fostering binding to different partners, atomic-level structural analyses of plectin’s diverse N-termini, including that of P1d, will be required. Unfortunately, this approach is hampered by some of the isoform-specific domains having an intrinsically disordered structure [48]. Once specifically targeted to Z-disks via HSC70, P1d can undergo interactions with a-actinin, sympo2, and likely other Z-disk-associated constituents via interfaces situated in molecular domains other than those forming the P1d-specific N-terminus. Such multiple interactions are expected to strengthen P1d’s affinity to Z-disk structures, thereby consolidating the compactness of IF-docking platforms along individual myofibrils and leading to their physical integration into the mechanotransducing IF network machinery.

Yeast two-hybrid screening revealed that P1d interacts with a wide spectrum of structural and signaling proteins. While its interaction with α-actinin appears to be crucial for the stabilization and lateral arrangement of Z-disks, P1d’s ability to bind to HSC70 and synpo2 suggested that its role goes beyond that of just mechanical support. Since HSC70 was identified in most of the positive transformants showing interaction with the short N-terminal region of P1d, one can assume that it is directly involved in targeting of plectin to the Z-disk. In contrast, α-actinin and synpo2 displayed binding to the longer fragment of P1d, suggesting that the binding site for these proteins is located in the region downstream of plectin’s ABD. This would be consistent with the previous observation that a fragment of the plakin domain binds to α-actinin (see above). Although α-actinin was clearly revealed as a P1d interaction partner using the yeast two-hybrid screening assay, additional in vitro binding studies showed that it can also bind to P1f. These observations imply that both plectin isoforms contain a binding site for α-actinin. While P1d interacts with α-actinin at the Z-disks and in this way stabilizes the contractile apparatus, P1f might be connected with cytoplasmic α-actinin residing underneath the sarcolemma where it supports membrane-associated protein complexes [11,12]. However, a specific N-terminus might modulate the remaining part of the molecule in a way that affects the binding affinity of interaction sites common to all isoforms, including the plakin domain-embedded α-actinin-binding site. Furthermore, plectin molecules can contain more than one binding site for a particular protein, as shown for β-dystroglycan [12]. To specify the location and structurally analyze the binding interfaces with distinct proteins, additional experiments including binding assays using truncated forms of plectin and atomic structure analyses need to be carried out.

Our study confirms that P1d deficiency causes disorganization and destabilization of Z-disks due to a disrupted IF network–contractile apparatus linkage and the formation of desmin aggregates accumulating in the fiber interior. However, as we show in the present report, P1d plays a role that goes beyond simple structural support by forming a scaffolding platform for the protein degradation machinery CASA. The increased filamin C protein levels observed in P1d-KO and MCK/Cre-cKO myofibers suggested that P1d-mediated homogeneity of Z-disk alignment is crucial for the proper functioning of the CASA machinery. Although many questions regarding the mechanism how P1d regulates CASA remain open, we can conclude from our data that (i) the efficient degradation of filamin C via CASA requires the presence of P1d, and (ii) P1d is crucial for the proper function of the protein degradation machinery that plays an important role in muscle maintenance, especially during intensive contraction. As misfolded filamin C is efficiently degraded and replaced by newly synthesized protein, filamin C is a mechanosensing protein which maintains the structural integrity of the myofibers and preserves the contractile apparatus during muscle contraction. Our analysis shows that the proper alignment of Z-disks seems to be especially important for the efficient removal of unfolded filamin C, as highly upregulated filamin C levels were observed in P1d-KO and MCK-Cre/cKO myofibers. P1d’s interaction with synpo2 suggests that P1d, functioning as a Z-disk-associated scaffolding platform, links the CASA machinery and the autophagic degradation pathway, while P1d-deficiency causes a misalignment of Z-disks and a shift and misplacement of CASA components. Measuring the mechanical strain exerted on cultured myotubes on a stretching device convincingly demonstrated that CASA is a tension-induced protein degradation machinery. As the cell stretcher used for these experiments could indeed mimic the naturally occurring skeletal muscle contraction, it could be used as a convenient tool to analyze multiscale mechanical responses governed by cytoskeletal prestress in ex vivo cell culture systems. Due to the disrupted HSC70-synpo2-Z-disk linkage and the ensuing dysfunction of CASA in plectin (P1d) deficiency, filamin C levels drastically increased upon mechanical strain in *Plec*^−/−^ myotubes (see Figure 6E). Moreover, unfolded filamin C was not degraded properly during muscle contraction, but instead accumulated in the form of massive aggregates which further destabilized the internal structure of the myofiber. The forced swim test experiment, showing a striking increase in filamin C aggregate formation and Xin-positive sarcomeric lesions, together with the increased amounts of filamin C upon stretch in *Plec*^−/−^ myotubes, indicated that the role of P1d becomes particularly important during intense mechanical strain. Considering that P1d-KO mice became exhausted much faster than wild-type mice, and that many of them died during the experiment, it remains to be tested whether they show any cardiac phenotype, and heart failure was the reason for their frequent death. Future experiments, especially resistance-based evaluations of muscle function in mice or using ex vivo whole muscle preparations in a fatigue protocol, would significantly add to our understanding of the role of P1d in mechanically-induced muscle damage.

## 5. Conclusions

In conclusion, our study shows that the Z-disk-associated plectin isoform P1d is indispensable for proper skeletal muscle function and maintenance, especially upon elevated tension and contractile activity. By interacting and encircling individual Z-disks, P1d bridges the contractile apparatus to the extrasarcomeric desmin IF cytoskeleton, thereby achieving the precise lateral alignment of sarcomeres required for the homogeneous spatial arrangement of myofibrils within myofibers. Aside from its structural role, P1d serves as a scaffolding platform for CASA, the tension-induced proteolytic machinery which becomes activated upon high loads of mechanical exercise and muscle contraction. Loss of P1d leads to the disorganization of myofibrils, combined with distortion of CASA and filamin C homeostasis, eventually manifesting as muscular lesions and protein aggregate formation. Our study offers a possible mechanistic explanation for the symptomatic muscle weakness associated with the majority of plectinopathies.

## Figures and Tables

**Table 1:** List of primary antibodies used for immunofluorescence microscopy (IFM) and/or immunoblotting (IB).

Protein	Source	Reference	Host and Clonality	Dilution
α-Actinin	Sigma-Aldrich	EA-53	Mouse mAbs ^1^	1:700 (IFM), 1:2000 (IB)
Plectin	G. Wiche	#46 [17]	Rabbit AS ^2^	1:100 (IFM)
Desmin	Dako	Clone D33	Mouse mAbs	1:100 (IFM)
HSC70	Abcam	ab-19136	Rat mAbs	1:100 (IFM), 1:2000 (IB)
Synpo2	D. Fürst	HH9 [18]	Mouse mAbs	1:1000 (IB)
Synpo2	D. Fürst	M2/1594 [18]	Affinity-purified isoform-specific rabbit AS	1:200 (IFM)
GST	Sigma-Aldrich	G1160	Mouse mAbs	1:1000 (IB)
HIS	Qiagen	6xHis-tag	Mouse mAbs	1:1000 (IB)
GAPDH	Sigma-Aldrich	G9545	Rabbit AS	1:5000 (IB)
Filamin C	D. Fürst	d16-20 [19]	Rabbit AS	1:1000 (IFM), 1:10,000 (IB)
BAG3	Proteintech	10599-1-AP	Rabbit AS	1:5000 (IB)
P62/SQSTM1	Sigma-Aldrich	P0067	Rabbit AS	1:1000 (IB)
Xin	D. Fürst	[20]	Mouse mAbs	1:100 (IFM)

^1^ mAbs, monoclonal antibodies; ^2^ AS, antiserum.

## Data Availability

The data presented in the study are available on request from the corresponding author.

## Supplementary Materials

The following supporting information can be downloaded at: https://www.mdpi.com/2073-4409/12/9/1259/s1, Figure S1: Original immunoblots for Figure 4C with molecular weight markers indicated; Figure S2: Original immunoblots for Figure 4D with molecular weight markers indicated; Figure S3: Original immunoblots for Figure 4E with molecular weight markers indicated; Figure S4: Original immunoblots for Figure 4F; Figure S5: Original immunoblots for Figure 4G; Figure S6: Original immunoblots for Figure 4J showing the bands with molecular weight markers; Figure S7: Original immunoblots for Figure 5A showing the bands with molecular weight markers; Figure S8: Original immunoblots for Figure 6A showing the bands with molecular weight markers; Figure S9: Original immunoblots for Figure 6D with molecular weight markers indicated.
