# Correction: Winter et al. Z-Disk-Associated Plectin (Isoform 1d): Spatial Arrangement, Interaction Partners, and Role in Filamin C Homeostasis. *Cells* 2023, *12*, 1259

**DOI:** 10.3390/cells12232677

**Published:** 2023-11-22

**Authors:** Lilli Winter, Ilona Staszewska-Daca, Stefan Zittrich, Fatiha Elhamine, Michaela M. Zrelski, Katy Schmidt, Irmgard Fischer, Christian Jüngst, Astrid Schauss, Wolfgang H. Goldmann, Robert Stehle, Gerhard Wiche

**Affiliations:** 1 Department of Biochemistry and Cell Biology, Max Perutz Laboratories, University of Vienna, 1030 Vienna, Austria; lilli.winter@meduniwien.ac.at (L.W.);; 2 Division of Cell and Developmental Biology, Center for Anatomy and Cell Biology, Medical University of Vienna, 1090 Vienna, Austria; 3 Institute of Vegetative Physiology, Medical Faculty, University of Cologne, 50931 Cologne, Germany; 4 Core Facility for Cell Imaging & Ultrastructure Research (CIUS), University of Vienna, 1030 Vienna, Austria; 5 CECAD Imaging Facility, CECAD Forschungszentrum Cologne, 50931 Cologne, Germany; 6 Department of Physics, Center for Medical Physics and Technology, Friedrich-Alexander-University Erlangen-Nuremberg, 91052 Erlangen, Germany

The authors wish to make the following changes to their paper [1]. In the original publication (pdf), there were mistakes in the legend for Figure 3 and in some text. Several lines of the legend were deleted, and one false line was inserted instead. Intermediate filaments were erroneously abbreviated as “Ifs” twice (each) in the Abstract, Introduction, and Discussion. It should read IFs throughout the text. 

The correct legend appears below:

**Figure 3.** Partial desmin IF collapse, sarcomere inhomogeneity, and altered relaxation kinetics of isoform P1d-deficient myofibrils. (**A**) Immunofluorescence microscopy of teased EDL muscle fibers from wild-type and plectin isoform P1d-deficient (P1d-KO) mice using anti-desmin- (**upper panels**) and anti-α-actinin-specific (**bottom panels**) antibodies. Nuclei are visualized using Hoechst staining in the upper panels. Note the massive accumulation of desmin-positive protein aggregates in the interior of P1d-KO fibers at the level of Z-disks (arrowheads) and the preserved perinuclear desmin staining pattern (arrows). Scale bars: 10 µm. (**B**) Electron micrographs of longitudinal soleus sections showing disturbed Z-band alignment and myofibrillar phase displacement in P1d-KO muscles. Scale bars: 1 µm. (**C**) Picture of a myofibril bundle mounted in the mechanical setup for force measurement. (**D**) Myofibril bundles isolated from P1d-KO mice were immunolabeled using antibodies to plectin. Note that hardly any plectin signals were obtained in P1d-KO myofibrils. Scale bar: 10 µm. (**E**) Statistical evaluation of the sarcomere length of myofibrils isolated from wild-type and P1d-KO mice. Mean ± SEM (wild-type (n = 19), MCK-Cre/cKO (n = 20); *N* = 3). (**F**) Statistical evaluation of the passive tension of wild-type and P1d-KO myofibrils. Mean ± SEM (wild-type (n = 15), MCK-Cre/cKO (n = 21); *N* = 3). (**G**–**J**) Ca^2+^-induced force development of wild-type and P1d-deficient myofibrils was assessed by switching from relaxing solution (pCa 7) to activating solution (pCa 4.5), yielding the maximum Ca^2+^-activated tension F_max_ (shown normalized to cross-sectional area of myofibrils in (**J**)) and the rate constant *k*_ACT_ (**H**). The rate constant *k*_TR_ was derived from the force redevelopment induced by a release–restretch maneuver applied to the bundle during Ca^2+^ activation (**I**). Mean ± SEM ((**H**) wild-type (n = 15), MCK-Cre/cKO (n = 22); (**I**) wild-type (n = 14), MCK-Cre/cKO (n = 21); (**J**) wild-type (n = 15), MCK-Cre/cKO (n = 22); *N* = 3). (**K**) Switching to Ca^2+^-free solution leads to a biphasic relaxation that was fitted by a biphasic function (see Methods section) yielding the rate constant of the slow phase (*k*_LIN_, (**L**)), the duration of the slow phase (*t*_LIN_, (**M**)), and the rate constant of the fast phase (*k*_REL_, (**N**)) of force decay. Mean ± SEM ((**L**–**N**) wild-type (n = 13), MCK-Cre/cKO (n = 19); *N* = 3). (**O**–**S**) Protocol to test mechanical stability of myofibrils against eccentric contractions during Ca^2+^ activation (**O**), statistical evaluations of the force reduction (**P**), the rate constant of the slow phase (*k*_LIN_, (**Q**)), the duration of the slow phase (*t*_LIN_, (**R**)), and the rate constant of the fast phase (*k*_REL_, (**S**)) after eccentric contraction. Note that, in general, relaxation kinetics were slower after the eccentric contraction protocol in myofibril bundles from wild-type and P1d-KO mice (dashed lines in (**Q**–**S**) represent values of respective parameters before the eccentric protocol, as shown in (**L**–**N**)). Also note that P1d deficiency enhances this effect, as the duration of the slow phase was significantly prolonged and the rate constant of the fast phase was significantly reduced after eccentric exercise in P1d-KO compared to wild-type myofibrils. Mean ± SEM ((**P**) wild-type (n = 15), MCK-Cre/cKO (n = 21); (**Q**–**S**) wild-type (n = 13), MCK-Cre/cKO (n = 19); *N* = 3). * *p* < 0.05 compared with wild-type, *t*-test.

The authors state that the scientific conclusions are unaffected. This correction was approved by the academic editor. The original publication has also been updated.
