# Supplementary material for: Z-Disk-Associated Plectin (Isoform 1d): Spatial Arrangement, Interaction Partners, and Role in Filamin C Homeostasis
Source: Cells. 2023 Apr 26;12(9):1259. doi: 10.3390/cells12091259 (PMC10177080; doi:10.3390/cells12091259)

Supplementary Material

Supplementary Figure S1. Original immunoblots for Figure 4C with molecular weight markers indicated.

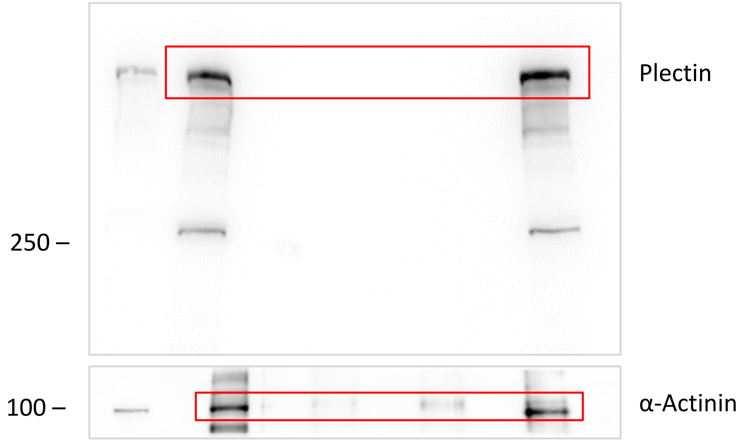

Supplementary Figure S2. Original immunoblots for Figure 4D with molecular weight markers indicated.

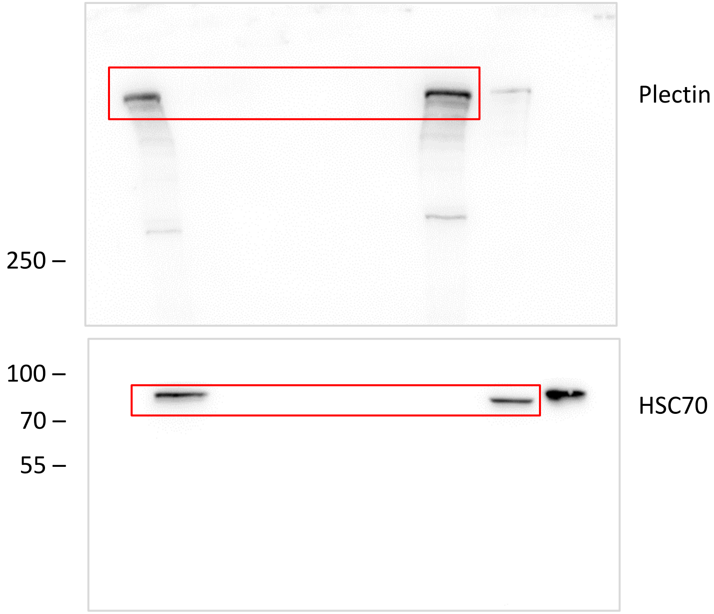

Supplementary Figure S3. Original immunoblots for Figure 4E with molecular weight markers indicated.

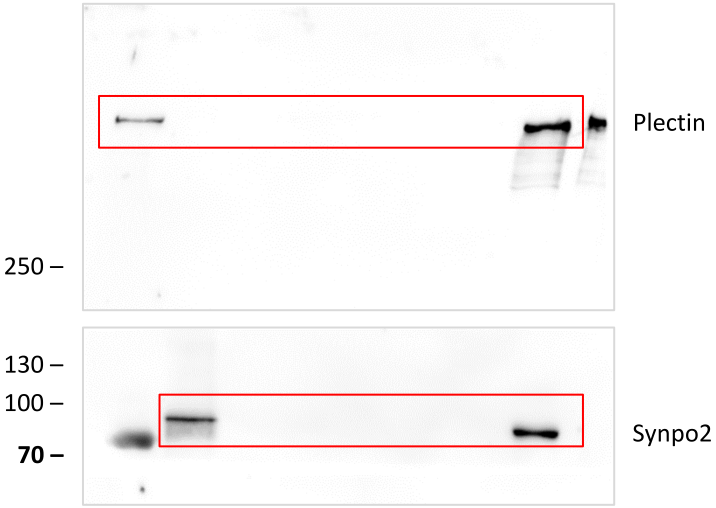

Supplementary Figure S4. Original immunoblots for Figure 4F.

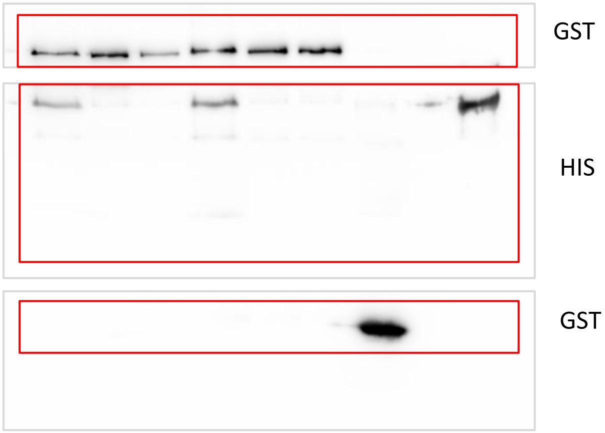

Supplementary Figure S5. Original immunoblots for Figure 4G.

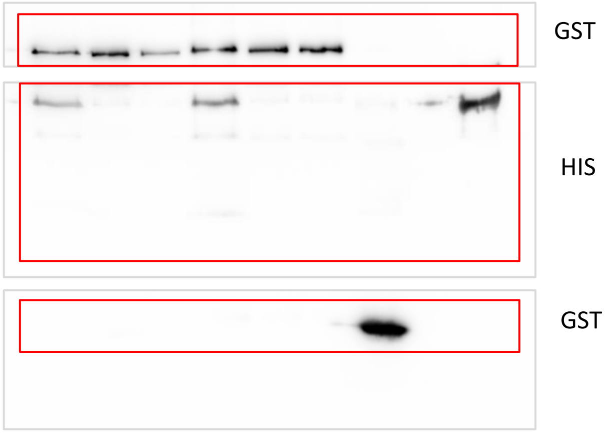

Supplementary Figure S6. Original immunoblots for Figure 4J showing the bands with molecular weight markers.

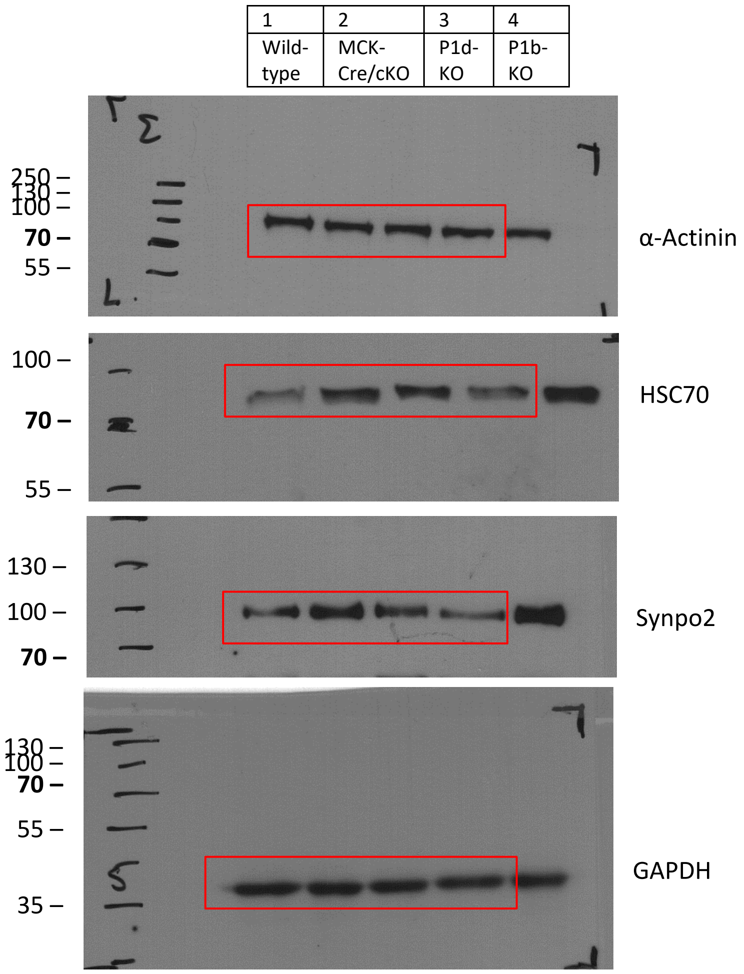

Supplementary Figure S7. Original immunoblots for Figure 5A showing the bands with molecular weight markers.

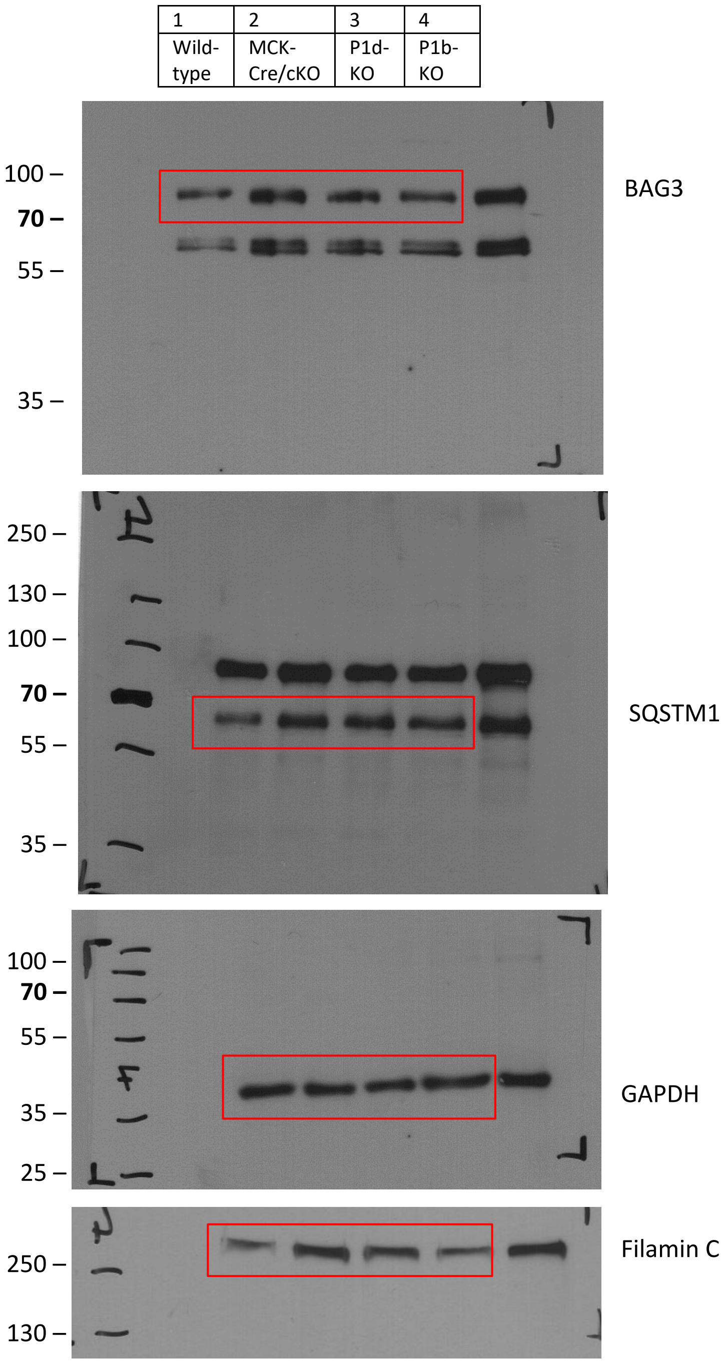

Supplementary Figure S8. Original immunoblots for Figure 6A showing the bands with molecular weight markers.

	1	2	3	4	5	6	7	8
Plectin	+/+	-/-	+/+	-/-	+/+	-/-	+/+	-/-

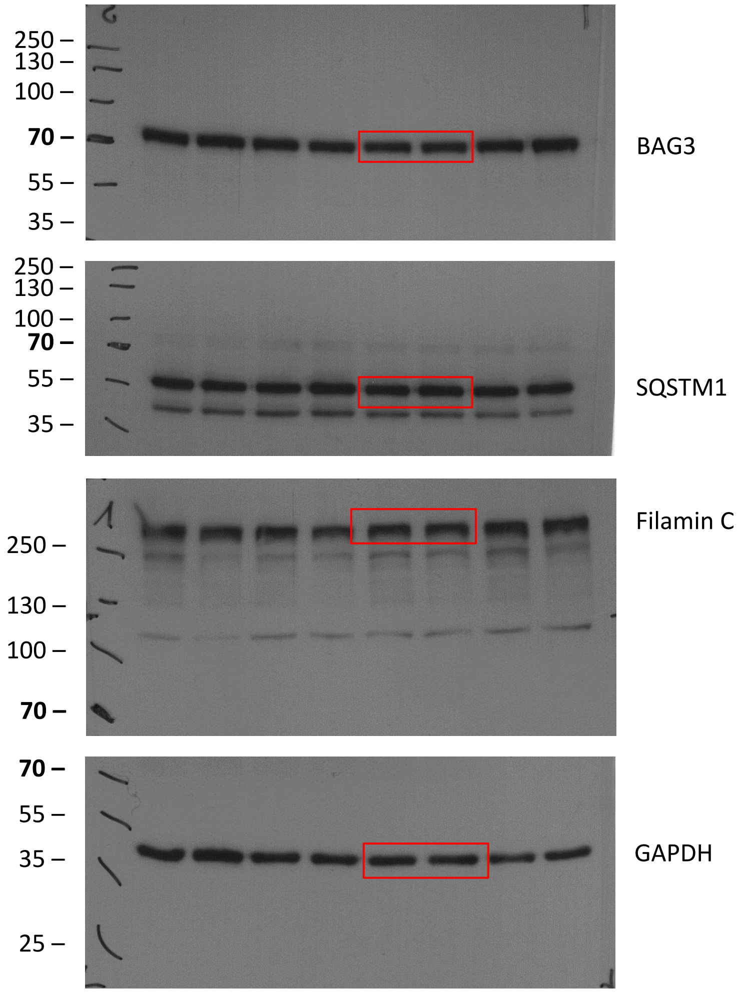

Supplementary Figure S9. Original immunoblots for Figure 6D with molecular weight markers indicated.

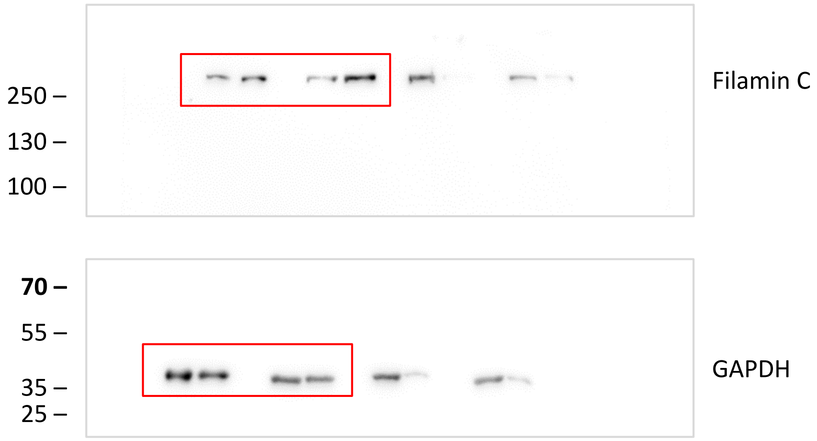

Supplement: Supplementary file 1 [file cells-12-01259-s001.zip › cells-2348815-supplementary.pdf]
